# A systematic review of approaches to assess fish health responses to anthropogenic threats in freshwater ecosystems

**DOI:** 10.1093/conphys/coae022

**Published:** 2024-05-04

**Authors:** Maxwell C Mallett, Jason D Thiem, Gavin L Butler, Mark J Kennard

**Affiliations:** Australian Rivers Institute, School of Environment and Science, Griffith University, 170 Kessels Road, Nathan, QLD 4111, Australia; New South Wales Department of Primary Industries, Narrandera Fisheries Centre, 70 Buckingbong Road, Narrandera, NSW 2700, Australia; New South Wales Department of Primary Industries, Grafton Fisheries Centre,16 Experiment Farm Road, Trenayr, NSW 2460, Australia; Australian Rivers Institute, School of Environment and Science, Griffith University, 170 Kessels Road, Nathan, QLD 4111, Australia

**Keywords:** Anthropogenic threats, conservation physiology, health indicators

## Abstract

Anthropogenic threats such as water infrastructure, land-use changes, overexploitation of fishes and other biological resources, invasive species and climate change present formidable challenges to freshwater biodiversity. Historically, management of fish and fishery species has largely been based on studies of population- and community-level dynamics; however, the emerging field of conservation physiology promotes the assessment of individual fish health as a key management tool. Fish health is highly sensitive to environmental disturbances and is also a fundamental driver of fitness, with implications for population dynamics such as recruitment and resilience. However, the mechanistic links between particular anthropogenic disturbances and changes in fish health, or impact pathways, are diverse and complex. The diversity of ways in which fish health can be measured also presents a challenge for researchers deciding on methods to employ in studies seeking to understand the impact of these threats. In this review, we aim to provide an understanding of the pathway through which anthropogenic threats in freshwater ecosystems impact fish health and the ways in which fish health components impacted by anthropogenic threats can be assessed. We employ a quantitative systematic approach to a corpus of papers related to fish health in freshwater and utilize a framework that summarizes the impact pathway of anthropogenic threats through environmental alterations and impact mechanisms that cause a response in fish health. We found that land-use changes were the most prolific anthropogenic threat, with a range of different health metrics being suitable for assessing the impact of this threat. Almost all anthropogenic threats impacted fish health through two or more impact pathways. A robust understanding of the impact pathways of anthropogenic threats and the fish health metrics that are sensitive to these threats is crucial for fisheries managers seeking to undertake targeted management of freshwater ecosystems.

## Teaser

The mechanisms through which human modification of freshwater environments impacts individual fish health are diverse and complex. We present a systematic review of research spanning 50 years to identify impact pathways of freshwater human-induced threats and the health assessment methods that are sensitive to them.

## Introduction

**Figure 1:**
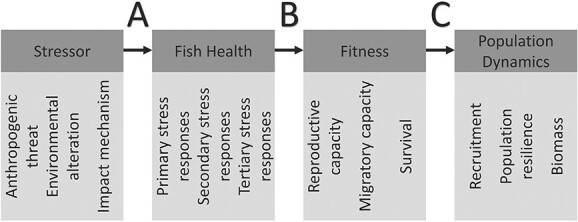
A conceptual model showing the sequential steps through which stressors impact fish health, fitness and population dynamics. ‘Stressor’ refers to anthropogenic threats and their resulting environmental alterations and impact mechanisms (described further on) that operate in freshwater environments. ‘Fish Health’ refers to health components that make up the primary, secondary and tertiary stress responses. ‘Fitness’ refers to parameters including but not limited to reproductive capacity, migratory capacity and survival. ‘Population dynamics’ refers to factors such as recruitment, population resilience and biomass. The letters ‘A’, ‘B’ and ‘C’ highlight the links between these steps.

Freshwater biodiversity constitutes a valuable resource, providing important economic, scientific, aesthetic, cultural and recreational ecosystem services (Dudgeon *et al.*, 2006). Despite this, global freshwater ecosystems are some of the most poorly managed, with anthropogenic disturbances occurring simultaneously with natural environmental variability. While anthropogenic pressures on freshwater ecosystems continue to mount globally, there are proportionally few studies that investigate the impacts of these pressures on individual fish health (Bergman *et al.*, 2019). Recent studies and reviews (e.g. Watson *et al.*, 2020; Brosset *et al.*, 2021) have described how particular stressors can impact physiological parameters with consequences for fitness and overall population dynamics. Here, we develop a conceptual framework that links stressors, fish health, fitness and population dynamics (Fig. 1). Research into the link between anthropogenic threats and individual fish health (Link A, Fig. 1) is limited, when compared to the body of research related to understanding the consequences of fish health for individual fitness, population dynamics and higher levels of ecological organization (assemblages and communities) and the study of their interrelationship (links B and C, Fig. 1) (Birk *et al.*, 2020; Brosset *et al.*, 2021; Pinna *et al.*, 2023).

Despite this, the importance of individual health is increasingly being recognized in conservation management, and the discipline of conservation physiology has emerged as a new science (Cooke *et al.*, 2022). Conservation physiology aims to use various physiological health components to understand how both natural and anthropogenic disturbances (or a combination of both) can translate into population level changes (Cooke *et al.*, 2013). In this context, physiological health components refer to cellular, organ and organismal function, including aspects such as behaviour. The potential utility of these health components as diagnostic tools is evident for several reasons. First, physiological fish health components can be highly sensitive to anthropogenic disturbances, and changes in health components are measurable well in advance of demographic changes, potentially affording sufficient time to implement threat mitigation strategies (Ellis *et al.*, 2012). Second, physiological indicators have a direct link to drivers of population dynamics such as individual fitness (Bergman *et al.*, 2019). Finally, the assessment of physiological health components can provide a robust and mechanistic understanding of the impact of anthropogenic stressors on an environment, as opposed to demographic indicators such as abundance or biomass (Horodysky *et al.*, 2015). Despite the apparent benefits of this discipline, the application of physiological biomarkers as management tools has only recently been applied to conservation challenges (Cooke and O’Connor, 2010; Madliger *et al.*, 2016; Bergman *et al.*, 2019).

In this review, we aim to provide an understanding of the initial pathway through which anthropogenic threats in freshwater ecosystems impact fish health, and synthesize the ways in which fish health components impacted by anthropogenic threats have been assessed as health indicators. We analyse a corpus of papers that assess the impact of multiple anthropogenic threats (e.g. water infrastructure, climate change, overexploitation, invasive species and land-use changes) on freshwater fish health. Using a quantitative systematic approach, we also quantify spatial and temporal trends within our corpus.

##  

### What is fish health?

Fish health comprises multiple physiological and behavioural components, each of which operates on different biological and temporal scales. Selected components of fish health are responsible for maintaining homeostasis—the maintenance of molecular, cellular and physiological function within a ‘normal’ range (Segner *et al.*, 2012; Yıldız and Seçer, 2017; Shiry *et al.*, 2023). The maintenance of homeostasis in turn enables other components of fish health to operate, such as growth to maintain condition, reproduction and locomotion (McEwen, 2000; Segner *et al.*, 2012; Lloret *et al.*, 2013; Sokolova, 2013; Schinegger *et al.*, 2016; Yıldız and Seçer, 2017; Balasch and Tort, 2019).

One way of summarizing components of fish health and how they interact with the environment is the categorization of responses to stressors (Wendelaar Bonga, 1997). Stress responses can be categorized into three main groups: primary stress responses, secondary stress responses and tertiary stress responses (Mazeaud *et al.*, 1977; Wedemeyer and McLeay, 1981; Barton *et al.*, 2002; Portz *et al.*, 2006; Segner *et al.*, 2012) (Fig. 1). Each level of stress response is comprised of different components that can be measured as a health indicator to infer overall fish health. Summarizing fish health in such a way helps in understanding that different components are sensitive to different types of stressors and respond on different time scales. Primary stress responses involve changes in neuroendocrine function, resulting in the release of hormones such as cortisol (Pottinger, 2008; Sheriff *et al.*, 2011). These hormones are released into the blood, where they can be measured to assess responses to stressors and initiate other physiological responses that make up the secondary stress response (Barton, 2002). Secondary stress responses primarily include changes in metabolic function, changes in immune system function, changes in haematological features and cellular changes such as increased heat shock proteins (HSPs) (Barton *et al.*, 2002), each of which are measurable as health indicators. Secondary stress responses may temporarily tolerate acute stressors and allow a return to homeostasis; however, if the stressor is severe or occurs for a period of time outside of regulatory capacity, primary and secondary stress responses may culminate in tertiary stress responses (Sopinka *et al.*, 2016). These stress responses are defined as whole-animal changes and include morphological changes such as decreased condition (Segner *et al.*, 2012). Condition is another health component used as a surrogate fish health indicator (Brosset *et al.*, 2023). There are also other methods that can be used to assess fish health that do not fall within these definitions. For example, the presence of parasites does not fall into these categories, as they can occur irrespective of physiological processes and health status. Details of commonly measured stress responses are provided in Table 1. A more detailed description of stress responses is provided in Supplementary Material S1.

**Table 1:** A summary of commonly measured primary, secondary and tertiary stress responses used as health indicators. Genetic indices and assessments of parasites and disease have been included as ‘other’

Health indicator	Field appropriate	Expertise required	Time required	Lethality	Cost	Relevant stressors	Key references
Primary stress responses							
Corticosteroid hormones. A range of hormones (including cortisol) released into the blood by the hypothalamus, pituitary gland and interrenal tissue (collectively known as the HPI axis).	Corticosteroid collection and measurement usually requires controlled laboratory methods. Faecal steroid hormone samples can be successfully collected in the field and transported to the laboratory.	Requires an experienced operator for sample collection and preparation.	Comparatively large amount of time required.	Can be a non-lethal indicator with correct methodology.	Comparatively high cost associated with laboratory analysis, compared to other stress responses.	Acute stressors such as handling and predation.	Barton *et al.*, (2002); Turner *et al.* (2003), Sadoul and Geffroy (2019)
Secondary stress responses							
Haematological assessments. Most commonly measured indices include metabolites such as glucose and lactate levels, blood acid–base properties, haematocrit and leukocrit and haemoglobin.	Field kits available for plasma glucose assessments however these require laboratory calibration.	Clinical test kits for measuring haematological parameters such as glucose and lactate are advantageous due to their ease of use.	Clinical test kits allow for quick assessment of haematological parameters such as glucose and lactate.	Non-lethal sampling method.	Haematological assessments range in cost from inexpensive field kits for plasma glucose assessment to expensive assessments such as transcriptomics.	Handling, temperature variation, abiotic factors (e.g. salinity).	Barton *et al.* (2002); Stoot *et al.* (2014); Seibel *et al.* (2021)
Oxidative stress. The transformation of oxygen to reactive oxygen species, with damaging effects involving structural modification of lipids, proteins and nucleic acids such as DNA.	Samples can be collected in the field for laboratory processing.	Sample processing requires laboratory experience.	Comparatively large amount of time required for laboratory analyses.	Oxidative stress biomarkers are often measured from organ tissue, necessitating organism death.	Tests for catalase, one of the most common biomarkers of oxidative stress, are relatively low cost.	The assessment of oxidative stress is commonly used in studies assessing the impact of heavy metal toxicity.	Gorbi and Regoli (2003); Viarengo *et al.* (2007); Kumari *et al.* (2014); García-Medina *et al.* (2022)

(*Continued*)

**Table 1:** Continued

Health indicator	Field appropriate	Expertise required	Time required	Lethality	Cost	Relevant stressors	Key references
Heat shock proteins (HSPs). Production of HSPs occurs through changes in gene expression HSPs are generally responsible for refolding denatured proteins, aiding in folding new proteins and deconstructing irreparable proteins.	Samples can be collected in the field for laboratory processing.	Specific experience and equipment required.	Common laboratory processes for HSP assessment are time consuming and not recommended for large sample sizes.	Methods exist to measure heat shock proteins in blood samples from living organisms.	Costs associated with laboratory analysis, compared to other stress responses.	Parasites, pollution, hypoxia, ultraviolet radiation, viral infection as well as hypothermia and heat shock can induce the production of HSPs	Feder and Hofmann, (1999); Liu *et al.* (2019); Njemini *et al.* (2020)Szyller and Bil-Lula (2021)
Osmotic and ion regulation. An important regulatory process that maintains correct concentrations of ions such as Na+ and Cl− and allows for the excretion of toxicants.	Point of care devices exist for measurement of a variety of ions.	High level of expertise required and specialized equipment.	Moderately time-consuming.	Methods exist to measure ions in blood samples from living organisms.	Costs associated with laboratory analysis.	Acute stressors such as handling and pollution.	Eddy (1981); Wendelaar Bonga and Lock (1992); Hoffmayer and Parsons (2001); Skomal and Mandelman (2012); Stoot *et al.* (2014); Sopinka *et al.* (2016)
Tertiary stress responses							
Morphological changes. Changes in length and weight, commonly referred to as condition. Assessed by numerous mathematical formulas.	Can be undertaken rapidly in a field setting.	Requires little prior experience and specific skills.	Minimal time required to take length and weight measurements.	Non-lethal and non-invasive.	Only requires basic inexpensive equipment (e.g. electronic balance, length measurement board).	Changes in condition can be sensitive to a broad range of stressors such as pollution, changes in hydrology and climate change.	Rennie *et al.* (2010); Hubená *et al.* (2020); Beesley *et al.* (2021)
Organosomatic changes. Changes in the mass of organs including the liver, spleen, gonads and ovaries, measured in proportion to overall body mass.	Can be undertaken in a field setting with suitable equipment.	Moderate expertise required for identification and extraction of various organs.	Moderate amount of time required compared to other condition indices (e.g. morphological assessments).	A lethal measure of condition.	Only requires basic inexpensive equipment (e.g. electronic balance).	Can be sensitive to pollution.	Dogan and Can (2011); Hauser-Davis *et al.* (2012)

(*Continued*)

**Table 1:** Continued

Health indicator	Field appropriate	Expertise required	Time required	Lethality	Cost	Relevant stressors	Key references
Behaviour. Expression of certain behaviours such as avoidance, locomotion, feeding or reflexes in response to a stressor.	Can be undertaken in a field setting using movement tracking devices (e.g. radio tags).	High level of expertise required to implement in a field-based scenario. High level of expertise required to implement in a laboratory-based scenario.	Reflex behaviours such as operculum and mouth clamping can be observed rapidly. Movement and feeding behaviours require longer durations of study.	Non-lethal measure.	Requires moderately to highly expensive equipment in field- and laboratory-based environment.	Acute stressors such as light, handling and noise in aquaculture settings can cause changes in reflex impairment. Pollution can impact behaviours such as feeding behaviour and movement.	Weis *et al.* (2001); Davis (2010)
Lipids and fats. Lipid stores in multiple bodily tissues, assessed through biochemical or visual methods.	Visual assessments of coelomic fat can be undertaken with field dissection kits.	Moderate level of expertise required for visual assessments. High level of expertise required for laboratory processing methods.	Laboratory methods can be time-consuming	A lethal measure of condition.	Equipment required can be highly expensive with high labour costs.	Reductions in habitat availability, water quality parameters, pollution, parasite infection and fluctuating thermal and hydrological regimes.	Dehn and Schirf (1986); Shulman and Love (1999); Couturier *et al.* (2020); Beesley *et al.* (2021)
External abnormalities. Visually identifiable features on the exterior of a fish, such as lesions, tumours, deformities or injuries.	Easily undertaken in a field setting.	Little expertise required.	The assessment of external abnormalities can be rapidly undertaken.	The assessment of external abnormalities is non-lethal.	The assessment of external abnormalities requires little additional costs.	The presence of external abnormalities has been linked to impaired water quality due to high levels of pollution.	Schmitt *et al.* (1999); Benejam *et al.* (2008); Simon and Burskey (2016); Bunt and Jacobson (2021)
Other							
Parasites and Disease. The presence of external or internal macro and microscopic parasites, and diseases associated with various organs.	Rapid assessment of external parasitise easily undertaken in a field setting.	Moderate level of expertise required to identify presence external parasites. High level of expertise required to identify presence of internal parasites.	The assessment of external parasites can be rapidly undertaken by visual assessment.	Assessment of external parasites is a non-lethal method. Assessment of internal parasites requires dissection of internal organs.	Assessment of external parasites requires little additional costs.	The presence of parasites has been related to pollution and presence of heavy metals.	Kennedy (1997); Blanar *et al.* (2009); Vidal-Martinez *et al.* (2010); Tesfaye *et al.* (2017)
Genetic responses. Replication of genes in response to a stressor, assessed through measured quantities of RNA and DNA.	Samples can be collected in the field for laboratory processing.	High level of expertise required for sample analysis.	Large sample sizes can be processed relatively quickly in the lab utilising specific methodologies and equipment.	Genetic indices can be non-lethal depending on tissue sample required.	Equipment required can be highly expensive with high labour costs.	Pollution, water infrastructure.	Viarengo *et al.* (2007); Foley *et al.* (2016); Jeffries *et al.* (2021)

### Linking anthropogenic threats to fish health

**Figure 2:**
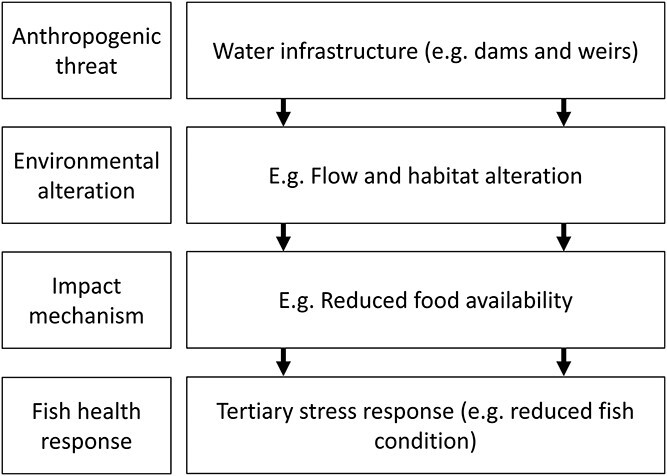
A conceptual diagram depicting our framework of an impact pathway, linking anthropogenic threats to environmental alterations, impact mechanisms and fish health responses. The anthropogenic threat ‘Water Infrastructure’ is used as an example, which leads to flow alterations resulting in changed food availability, impacting fish condition as a tertiary stress response.

To employ physiological biomarkers as effective management tools, it is important to understand how anthropogenic threats influence and modulate stress responses within an ecologically relevant framework (Cooke and O’Connor, 2010). Anthropogenic threats illicit a broad range of stress responses in fish and the mechanisms by which these threats ultimately impact fish health can be unclear (Sokolova, 2013; Schinegger *et al.*, 2016). Previous reviews have created hierarchical frameworks that step out these mechanisms. For example, Craig *et al.* (2017) define anthropogenic ‘threats’ as human-caused drivers of environmental change and environmental ‘alterations’ and ecological ‘effects’ as the environmental changes they produce and the ecological responses to those changes, respectively. Our review adopts a similar framework, however, the term ‘impact mechanism’ has been used instead of ecological ‘effects’ as our effects refer specifically to the mechanisms by which fish health can be directly impacted, and in some cases, these are not through changes in ecological function. We define unique combinations of anthropogenic threats, environmental alterations, impact mechanisms and health responses as an impact pathway. Figure 2 shows our impact pathway framework in use, with the anthropogenic threat ‘Water Infrastructure’ used as an example.

In our review, this framework is applied to the five major anthropogenic threats to freshwater ecosystems (Dudgeon, 2019). These anthropogenic threats are ‘Water infrastructure’, ‘Land-use changes’, ‘Invasive species’, ‘Overexploitation’ and ‘Climate change’. Water infrastructure pertains to the construction of dams, weirs and other barriers, resulting in environmental alterations including flow alteration (i.e. changes to the magnitude or timing of river flows), habitat alteration (loss of connectivity or critical habitat), abstraction, river impoundment and changed water quality (Fig. 2). Land-use changes refers to agriculturalization, industrialization and urbanization. Pollution is often considered as a broader anthropogenic threat; however in this review, it has been included as a sub-category of land-use (Tahiru *et al.*, 2020). Freshwater ecosystems are particularly vulnerable to pollution, with chemicals, heavy metals, microplastics and other particulate matter being of greatest concern (Bashir *et al.*, 2020; Gokul *et al.*, 2023). Other environmental alterations deriving from land-use changes include the removal of terrestrial vegetation and flow alteration. The introduction of invasive species into freshwater ecosystems is primarily a result of anthropogenic practices, either intentionally or unintentionally, leading to impact mechanisms such as increased competition, predation and the introduction of disease and habitat degradation. Overexploitation refers to the unsustainable use of freshwater resources, encompassing recreational and commercial over-fishing and the impacts of handling on both target species (e.g. catch-and-release recreational fishing) and bycatch (e.g. commercial bycatch returned to the water). Climate change acts as a ubiquitous threat to freshwater ecosystems and has the potential to exacerbate the effect of the above anthropogenic disturbances.

## Methods

### Literature search and database development

The review protocol followed that outlined by PRISMA (Preferred Reporting Items for Systematic Reviews and Meta-Analyses) (Supplementary Fig. S1) (Page *et al.*, 2021) and a ROSES (Reporting standards for Systematic Evidence Syntheses) form is included in Supplementary Sheet S1. While a systematic protocol was followed in the creation and categorization of our corpus, we refrain from asserting confidence in our control for validity and bias reduction, as we did not conduct critical appraisal of each paper to check for any biases in experimental design, data collection or analyses. The Clarivate Web of Science™ database was used to generate a corpus of 4397 papers. Defining the search terms was an iterative process, with different combinations of words being tested to generate the optimum corpus. The following final word combination was used: (Fish* AND (stress OR condition OR fitness OR health* OR fat* OR plump* OR (Condition AND Growth)) NOT (Oil* OR Chick* OR Rat* OR “conditions”)). Papers were filtered before export to only include those written in English over the last 50 years (1971–2021).

Refining the corpus further occurred as a two-stage process: Stage 1: screening and Stage 2: categorisation (Supplementary Fig. S1). The first stage involved rapid assessment of the title and abstract to check for relevancy, with all papers that measured fish health in some way retained. Papers that did not aim to measure fish health were excluded (e.g. papers that assessed fish palatability or the relation of fish health with the production of fish-based products for human consumption). Duplicate papers, grey literature, reviews and meeting abstracts were also removed during this stage, with a total of 2522 papers excluded from the corpus. During the second stage, the remaining 1875 papers were categorized to generate a dataset for the quantitative systematic literature review. A further 1514 papers were excluded as they did not assess responses in fish health to an anthropogenic disturbance, leaving a total corpus of 361 papers. Papers were then categorized by geographic location, study type (laboratory, field or a combination of both etc.), anthropogenic disturbance type (anthropogenic threats, environmental impacts and impact mechanisms, described above) and stress response. To categorize the anthropogenic disturbance type, the following definitions were used. *Anthropogenic threats* refer to the broad, basin scale threats that impact freshwater ecosystems*. Environmental impacts* refer to either the resulting changes in an environment that occur because of an anthropogenic threat (e.g. river impoundment as a result of water infrastructure) or a secondary threat as a result of an anthropogenic threat (e.g. fishing as a subgroup of over-exploitation). *Impact mechanisms* refer to the various mechanisms that have a direct impact on fish health components. For example, changed food availability can directly impact whole-body morphology, organosomatic indices or lipid and fat content.

Due to the large number of ways in which the primary and secondary stress responses can be measured, assessment methods belonging to these categories were grouped. Health components that belonged to the tertiary stress response, or health components considered to fall outside of primary, secondary or tertiary stress responses were individually categorized. Studies were also categorized by whether they were conducted in either an artificial setting, the field, a laboratory or a combination of field and laboratory settings. Laboratory studies included experiments on both living and dead fish. Field and laboratory studies were defined as studies that had both field and laboratory components. For example, the sampling of fish from different rivers where an attribute of the river (e.g. pollution) was predicted to have an impact on the result, but further laboratory analysis was required to obtain results. Field specific studies were those conducted entirely in the field, employing methods that did not require any further laboratory processing. Artificial studies consisted of studies either *ex situ*, or those conducted *in situ* in a separate enclosure. These studies were not classified as laboratory studies as they included the use of mesocosms or purpose-built enclosures where the physical size and shape of the tank were important to the study (e.g. simulating the presence of a fish screen or artificial sound/light). Further detail of the fish health categories used in this review is provided in Supplementary Table S1. The complete corpus and categorization criteria for this study are provided in Supplementary Sheet S2.

### Data analysis

#### Quantitative summary

The dataset generated from paper categorization was used to investigate: (i) the geographical distribution and temporal variation of studies within the corpus, (ii) the frequency of different study types, (iii) the prevalence of different fish health assessment methods used to evaluate responses to anthropogenic stressors and (iv) the impact pathways of anthropogenic threats that results in changes to fish health components. To quantify impact pathways, the anthropogenic threat discussed in each paper was broken down into three hierarchical categories: (i) the type of anthropogenic threat, (ii) environmental alterations (the way in which these anthropogenic threats alter the environment) and (iii) impact mechanisms, the mechanism by which environmental impacts cause changes in fish health indicators. Anthropogenic threat categories, environmental impacts and impact mechanisms were adopted from multiple reviews of anthropogenic threats in freshwater ecosystems (e.g. Dudgeon *et al.*, 2006; Dudgeon, 2019) and expert knowledge. These were then linked with the type of fish health indicator each study employed to understand the mechanism by which anthropogenic threats changed components of fish health. This information was presented as an alluvial diagram, a type of flow chart that can be used to represent nested hierarchical structures. For this visualisation, we utilized the ‘ggalluvial’ package (Brunson and Read, 2023) in R Studio (R studio version 4.3.0).

## Results

**Figure 3:**
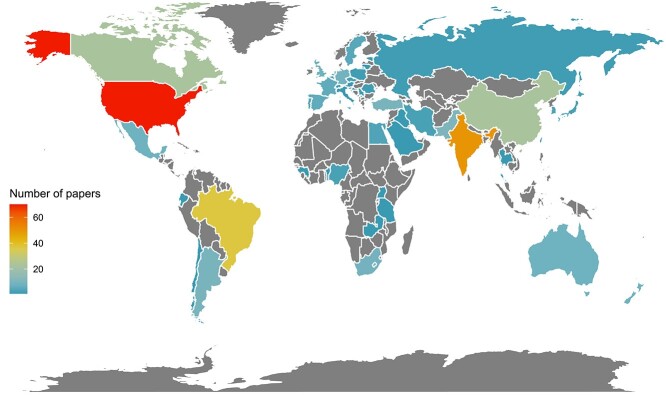
A global map showing the number of papers examining impacts of anthropogenic disturbances on fish health in freshwater systems published by each country. Grey polygons represent no papers published.

Papers that assessed the impact of anthropogenic disturbances on fish health in freshwater systems were derived from 49 countries. Of these countries, the most studies were undertaken in the United States and Canada (combined) (*n* = 93) (Fig. 3). Of the remaining 312 studies, 105 were from Asia, 73 were from Europe, 56 were from South America, 17 were from Africa and 16 were from Oceania (Australia and New Zealand).

**Figure 4:**
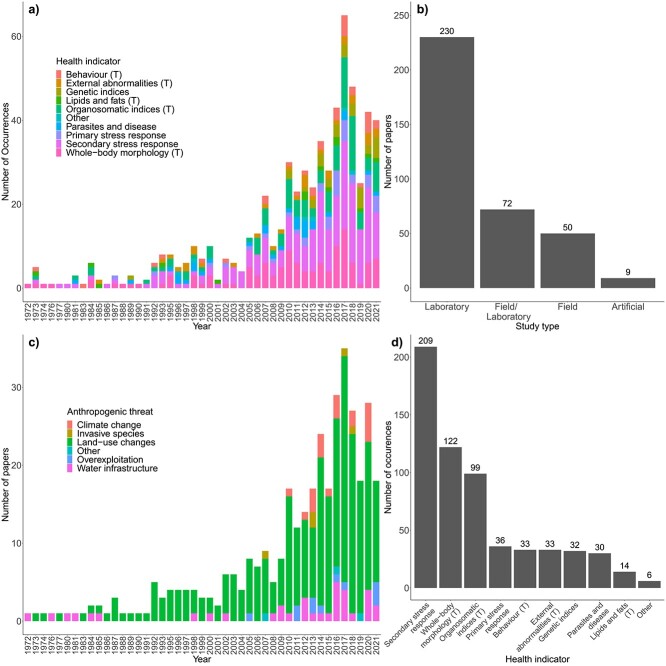
The frequency of occurrence of health indicators in our corpus from the years 1972 to 2021 (a), the number of studies that were conducted in an artificial, field, field/laboratory or laboratory setting (numbers above bars represent number of papers per study type) (b), the frequency of occurrence of anthropogenic threats in our corpus from 1972 to 2021 (c), and the number of occurrences of each health indicator used within the corpus (numbers above bars indicate number of occurrences per health indicator) (d). Tertiary stress responses are marked with a ‘T’ in panels (a) and (d). For panels (a) and (c), some papers within the corpus used multiple health indicators, meaning the number of occurrences shown in this figure is greater than the number of papers within the corpus.

We found that studies assessing the links between anthropogenic threats in freshwater ecosystems and fish health components have increased through time from 1972 to 2021 (Fig. 4a and c). The highest output of papers was in 2017, with 35 papers published. The last decade produced 66% of all papers included in the corpus. Our corpus primarily consisted of laboratory studies, with studies consisting of both field and laboratory components being the second most common (Fig. 4b). The combined total of field and artificial studies comprised less than a quarter of all studies within our corpus. Land-use changes (*n* = 288) were the most commonly studied anthropogenic threat and occurred in every year since 1986 (Fig. 4c). Climate change first occurred within our corpus in 2010 and is the newest threat to be studied in relation to fish health within the 21st century, comprising 19 studies in total. The impact of water infrastructure has been studied for the longest period of time out of all our threat categories. Other threats (‘Other’, Fig. 4c) included impacts of artificial noise, artificial light and the impacts of bushfires, with only a single study related to each of these threats over the study period. Secondary stress responses were the most common health indicator reported, totalling 209 instances across all years and occurring in 37 individual years (Fig. 4a and c). Tertiary stress responses including whole-body morphology (122 occurrences across 33 years) and organosomatic indices (99 occurrences across 28 years) were the second and third most common health indicator (Fig. 4a and c). Other health metrics included assessments of skin mucous, otolith asymmetry and electrocardiograms, and were the least common, with six combined occurrences over the study period.

**Figure 5:**
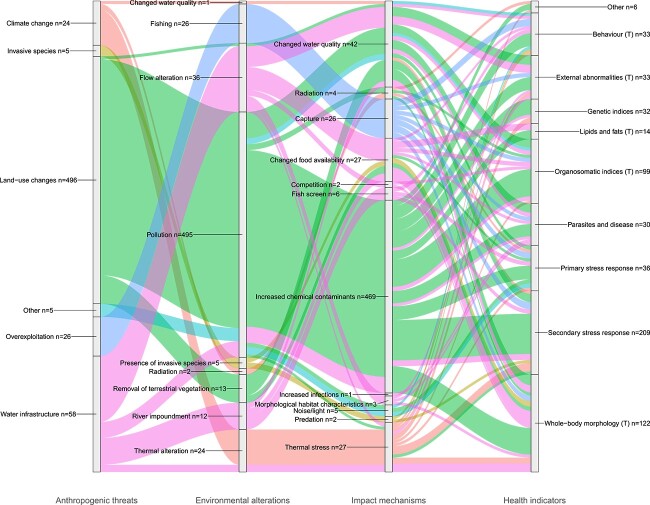
An alluvial diagram highlighting a network of four stratum: anthropogenic threats, environmental alterations, impact mechanisms and health indicators (tertiary stress responses denoted with T). The relative size of each category within each stratum is based on transformed (square root) occurrence counts to aid readability and is therefore not to scale. The plot is read either from left to right or right to left, with each stratum connected by ‘flows’, forming an 'impact pathway' across the four stratum. The proportion of each category within a stratum is provided numerically ‘*n*’.

**Figure 6:**
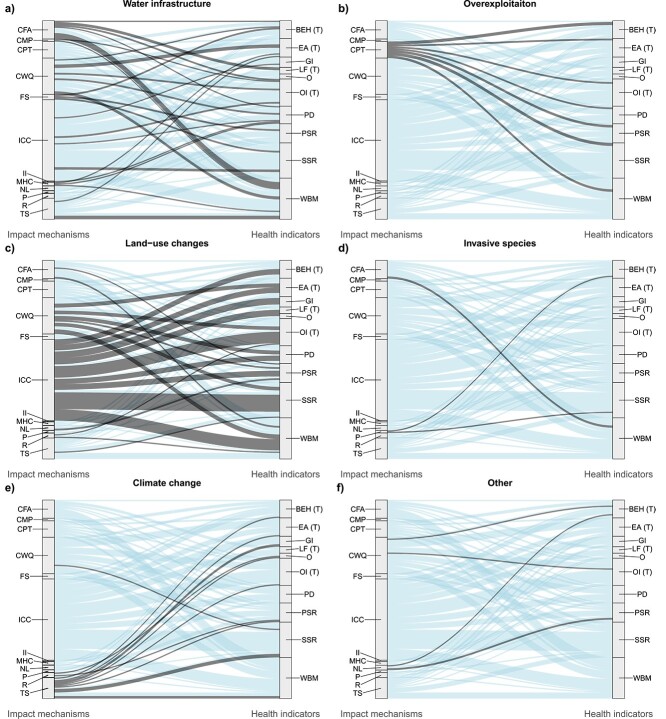
Six alluvial diagram subsets, one for each anthropogenic threat category from the main alluvial diagram in Fig. 5: (a) water infrastructure, (b) overexploitation, (c) land-use changes, (d) invasive species, (e) climate change and (f) other. Each subset depicts the last two stratum, impact mechanisms and health indicators, of the main alluvial diagram in order to highlight the final stage of the impact pathway of each anthropogenic threat. Impact mechanism abbreviations are as follows (from top to bottom): CFA = changed food availability, CMP = competition, CPT = capture, CWQ = changed water quality, FS = fish screen, ICC = Increased chemical contaminants, II = Increased infections, MHC = morphological habitat characteristics, NL = noise/light, P = predation, R = radiation and TS = thermal stress. Health indicator abbreviations are as follows (from top to bottom): BEH = behaviour, EA = external abnormalities, GI = genetic indices, LF = lipids and fats, O = other, OI = organosomatic indices, PD = parasites and disease, PSR = primary stress response, SSR = secondary stress response and WBM = whole-body morphology.

A total of 361 papers met the selection criteria for this review; however, a proportion of papers employed multiple health assessment methods, resulting in a total of 614 individual occurrences of fish health indicators. Figure 5 depicts these occurrences over a network of four stratum; *Anthropogenic threats*, *Environmental alterations*, *Impact mechanisms* and *Health indicators*, which when connected together by flows (coloured lines) form an impact pathway. Impact pathways can be followed either from left to right or right to left. Following from left to right uncovers the impact pathways a given anthropogenic threat follows to ultimately cause responses in fish health indicators. Following an impact pathway from right to left gives insight into the collections of threats that each health indicator is responsive to. Anthropogenic threats were dominated by land-use changes, largely due to a high proportion of papers relating to the impact of pollution from industrial, agricultural and urban inputs. As a result, increased chemical contaminants were the most common impact mechanism. In total, there were 81 unique impact pathways investigated within our corpus and despite land-use changes being the most common anthropogenic threat, water infrastructure resulted in the largest number of unique impact pathways (*n* = 29). Water infrastructure resulted in four different environmental impacts including thermal alteration, river impoundment, pollution and flow alteration. Land-use changes (*n* = 27) and climate change (*n* = 11) had the second and third highest number of unique impact pathways respectively. The threat of invasive species had the fewest unique impact pathways (*n* = 3), with the primary impact mechanisms being predation and changes in food availability, linked to only three health responses. These health responses can be seen in Fig. 6, which depicts the last two stratum of the main alluvial diagram for each anthropogenic threat to provide a clearer picture of the connections between impact mechanisms and health indicators. Health indicators responsive to the impact mechanisms resulting from invasive species were behaviour, secondary stress responses and whole-body morphology (Fig. 6d). Impacts of artificial noise and light (categorized as other anthropogenic threats) were assessed using behavioural and primary stress response indicators (Fig. 6f). Increased chemical contaminants, largely driven by land-use changes, were the only impact mechanism linked to every health indicator recorded (Fig. 6c). Behaviour was the only health indicator to be measured in response to all our anthropogenic threat categories. The health indicators measured in response to the fewest anthropogenic threats were ‘other’ health indicators including assessments of skin mucous and otolith chemistry (assessed in response to climate change and land-use changes), as well as the assessment of lipids and fats (land-use changes and water infrastructure).

## Discussion

This review compliments and extends on previous reviews (e.g. Brosset *et al.*, 2021; Gomez Isaza *et al.*, 2022; Stevenson and Woods, 2006) by focusing on the link between anthropogenic threats and individual fish health. By classifying papers based on a hierarchical framework of anthropogenic threats, environmental alterations, impact mechanisms and health indicators, we uncovered the impact pathways of six categories of anthropogenic threats to understand the mechanism by which these threats can impact fish health. We found that the impact pathways of these anthropogenic threats were complex. Multiple anthropogenic threats were related to the same environmental alterations and impact mechanisms. For example, studies related to the anthropogenic threats of water infrastructure and land-use changes both resulted in flow alteration (Fig. 6a and d). Overlapping of these anthropogenic threats leading to changes in hydrology has also been highlighted in previous studies and reviews (Poff *et al.*, 2006; Poff and Zimmerman, 2010). Craig *et al.* (2017) refer to impact pathways as alteration profiles and define similar alteration profiles as having two or more shared environmental alterations. In most freshwater ecosystems, multiple anthropogenic threats are likely to be operating in tandem, and this can lead to combined alterations that can produce either additive, antagonistic or synergistic effects on fish health (Crain *et al.*, 2008; Todgham and Stillman, 2013; Craig *et al.*, 2017). A number of studies had anthropogenic threats that shared little overlap in environmental alterations and impact mechanisms. For example, studies in our corpus related to overexploitation followed a unique impact pathway, impacting fish health through fishing and subsequent handling.

In some instances, anthropogenic threats were directly linked to fish health metrics. Several articles directly linked thermal changes resulting from climate change to changes in fish health metrics such as secondary stress responses (Fig. 5) (Templeman *et al.*, 2014; Kumar *et al.*, 2020). It is likely that the impacts of climate change also have more indirect impacts in freshwater environments (Dudgeon, 2019); however, this finding highlights that anthropogenic threats can directly impact organisms within an environment instead of through complex pathways associated with environmental alterations and impact mechanisms (Watson *et al.*, 2020).

Our corpus of articles was dominated by studies employing laboratory-based assessments of fish health. This is likely related to the large proportion of studies assessing the impacts of pollution on fish health. Health components sensitive to pollution are typically related to primary and secondary stress responses, and most of these methods require laboratory analyses (i.e. the assessment of glucocorticosteroids within blood and tissue, the assessment of HSPs and the assessment of oxidative stress biomarkers (Sopinka *et al.*, 2016). Contrary to our findings, in their review of the impacts of wildfires and associated runoff on aquatic fauna, Gomez Isaza *et al.* (2022) found that most studies they reviewed employed *in situ* (field-based) methods. Gomez Isaza *et al.* (2022) did not report on the types of health assessment methods that were attributed to *in situ* and laboratory studies; however, this difference in results suggests that the study type employed to assess the impacts of anthropogenic threats is highly dependent on the anthropogenic threat in question. In a review of management of wild fish populations in the Anthropocene, Cooke *et al.* (2022) highlight that field-based studies on a large number of individuals is likely the most valuable type of study for assessing responses in health to anthropogenic threats, and these types of studies are severely lacking within the literature. The comparatively low number of field-based studies in the corpus of this review further highlights this.

While our review focused solely on studies in the freshwater realm, many of the anthropogenic threats, their impact pathways and resulting fish health responses discussed in our corpus likely also operate in marine and transitional environments. In particular, Arthington *et al.* (2016) highlight overexploitation, climate change, the presence of invasive species and pollution as some of the most serious threats to marine fishes. These threats in marine and transitional environments can follow a multitude of direct and indirect impact pathways that mirror those in freshwater ecosystems (Schull *et al.*, 2023). For example, the impact of climate change resulting in increased thermal variation and extremes, causing primary and secondary stress responses, as well as the impact of land-use changes resulting in eutrophication, impacting a number of fish health components such as energetic reserves and immune response (Alfonso *et al.*, 2021; Schull *et al.*, 2023).

Finally, it is important to highlight that fish health responses can in some instances be sensitive to particular life stages and other intrinsic factors, meaning there are potential age- and size-specific considerations that should be made when investigating how fish respond to a stressor in question (Schull *et al.*, 2023). Indeed, particular fish health indicators may be better suited to studying impacts of stressors at specific life stages. For example, RNA:DNA ratios have been recommended for use on juvenile or small-bodied fishes where body size may prohibit the use of other condition indices (Foley *et al.*, 2016). Additionally, responses in fish health to stressors can be impacted by extrinsic abiotic features such as depth, salinity and other habitat characteristics (Schwartz *et al.*, 2004; Zhao *et al.*, 2013; Duarte *et al.*, 2018).

## Conclusion

Anthropogenic threats in freshwater environments pose a dynamic and complex problem; however, much of our knowledge relating to these impacts has focused on population- and community-level effects in freshwater ecosystems. Emerging fields such as conservation physiology seek to change this status quo and highlight the importance of individual organism health for overall population persistence and community stability. Our review identified key mechanistic links in the form of environmental alterations and impact mechanisms, through which anthropogenic threats impact fish health. Land-use changes were the most commonly studied anthropogenic threat. Anthropogenic threats impacted fish health through a diversity of impact pathways with water infrastructure consisting of the most unique combinations of impact pathways. We found that a large variety of fish health metrics are sensitive to these disturbances; however, secondary stress responses were the most commonly employed health indicator. We found that research related to freshwater fish health has been increasing over the past 50 years, with research dominated by laboratory studies. In light of our findings, future studies investigating anthropogenic impacts on individual fish health should seek to understand both the specific mechanisms and pathways through which anthropogenic threats may cause environmental alterations that impact fish health; as well as the fact that multiple anthropogenic threats can interact in unpredictable ways that can positively (additive or synergistic) or negatively (antagonistic) influence fish health. Linking threats to environmental alterations is crucial for ensuring effective management that addresses primary causes. We maintain that understanding drivers of individual fish health has practical implications for environmental managers, as individual fish health is the fundamental connection between anthropogenic threats and demographic changes at the population and community levels.

## Supplementary Material

Web_Material_coae022

## Data Availability

The data underlying this article are available in the article and in its online supplementary material.
