# Supplementary material for: A systematic review of approaches to assess fish health responses to anthropogenic threats in freshwater ecosystems
Source: Conserv Physiol. 2024 May 4;12(1):coae022. doi: 10.1093/conphys/coae022 (PMC11069195; doi:10.1093/conphys/coae022)
Supplement: Web_Material_coae022 [file web_material_coae022.zip › Supplementary material.pdf]

Supplementary Material 1

A summary of fish health components

The purpose of this section is to provide a brief structured description of fish health components and how changes in health components as a response to a stressor can be assessed. Changes to health components including physiological, genetic, morphological and behavioural components are categorised as primary, secondary and tertiary stress responses. This framework helps to step out the process by which an environmental stressor (e.g. anthropogenic threats in freshwater ecosystems) can act on multiple interconnected health components in a hierarchical and stepwise way, ultimately leading to changes in overall health that can have population and community implications.

Primary Stress Responses

The primary stress response can be considered as the starting point through which environmental stressors act on fish health components. This response is autonomous, occurring through the neuroendocrine system (Pottinger, 2008; Sheriff et al., 2011). The response operates via two main pathways (Sheriff et al., 2011). Firstly, the sympathetic nervous system, the part of the nervous system responsible for flight or fight adaptations (Sopinka et al., 2016), triggers the production of catecholamines such as epinephrine (Barton, 2002; Schulte, 2014). Secondly, the hypothalamus, pituitary gland and interrenal tissue (collectively known as the HPI axis) operate to release corticosteroids such as cortisol (Madaro et al., 2020). These hormones are released into the blood, where they initiate other physiological responses that make up the secondary stress response (Barton, 2002).

Assessment of the primary stress response is related to these two pathways. Activation of the sympathetic nervous system is typically indicated by measurement of catecholamines in plasma using chromatography with electrochemical detection (Woodward, 1982). The measurement of catecholamines is most often carried out in the lab, as their assessment can be logistically challenging in the field due to their high responsiveness to capture and handling (Sopinka et al., 2016). Out of the range of hormones released during the activation of the HPI axis, measurement of blood cortisol levels is one of the primary health components that studies use to measure the impact of stressors (Barton et al., 2002; Pottinger, 2008; Sadoul & Geffroy, 2019). Pottinger (2008) describes that a useful indicator of stress in fish would require a stable baseline from which changes could be measured. After a stressor occurs, there is a delay in the release of cortisol of several minutes (Barton, 2002). Barton (2002) describes that with proper sampling techniques, this delay allows for the measurement of resting cortisol levels, along with increased cortisol levels after a stress has been experienced. For a more detailed summary of techniques related to the assessment of primary stress response health components see Sheriff et al. (2011).

The measurement of health components that operate as part of primary stress responses lend themselves well to studies that aim to measure the impacts of acute stressors (Barton et al., 2002; Breuner et al., 2008; Sopinka et al., 2016). As the primary stress response operates on a time frame of minutes, they have limited applications in measuring the response of fish experiencing sublethal chronic stressors (Barton, 2002). In a study of the cortisol response of salmonids to an artificially fluctuating flow regime, Flodmark et al. (2002) found that although an initial fluctuation in flow triggered an elevation in cortisol levels, cortisol levels after cyclical fluctuations in flow for seven days had returned to normal. Flodmark et al. (2002) explain that a return to normal cortisol levels during chronic sublethal stressors could be due to the ability of the fish to either habituate to the

stressor or utilise a compensatory strategy such as moving between suitable habitats depending on the presence of the stressor.

Secondary Stress Response

The release of catecholamines and corticosteroids elicits a broad range of physiological responses which constitute the secondary stress response (Pottinger et al., 1994). Secondary stress responses comprise a number of different components of fish health, most commonly operating on the blood and tissue level (Skomal & Mandelman, 2012; Wendelaar Bonga, 1997). Secondary stress responses include haematological changes (Barton et al., 2002), oxidative stress responses (Chowdhury & Saikia, 2020), heat shock protein production (Roberts et al., 2010), osmolarity and ion concentrations (Wendelaar Bonga & Lock, 1992) and changes in immunity (Weyts et al., 1999). This review does not attempt to provide an in-depth description of each of these responses, and readers are instead directed to the cited literature. The type of secondary stress response that will occur is dependent on the magnitude and nature of the stressor, as well as the species-specific tolerance to that stressor (Chowdhury & Saikia, 2020; Skomal & Mandelman, 2012).

Description of Methods

Haematological assessments

The range of methods established for assessing blood as an indicator of fish health is now broader than for any other biogenic test material (Seibel et al., 2021). There are numerous haematological responses to stress, however the most commonly measured indices include metabolites such as glucose and lactate levels, blood acid-base properties, haematocrit and leukocrit (proportion of red and white blood cells in blood respectively) and haemoglobin (Barton et al., 2002; Skomal & Mandelman, 2012; Sopinka et al., 2016).

Oxidative Stress

Oxidative stress refers to the undesirable transformation of oxygen to reactive oxygen species (ROS) (Chowdhury & Saikia, 2020). Free-radical ROS species are those with one or more unpaired electrons, making them highly reactive with other molecules (Chowdhury & Saikia, 2020; Kroon et al., 2017). ROS have multiple damaging effects involving structural modification of lipids, proteins and nucleic acids such as DNA (Chowdhury & Saikia, 2020; Kumari et al., 2014). This in turn can lead to the activation of cell death processes such as apoptosis, as well as other pathological processes leading to various diseases (Redza-Dutordoir & Averill-Bates, 2016; Szyller & Bil-Lula, 2021). There are two main sources of oxidative stress; chemotoxicity induced oxidative stress and environmental induced oxidative stress. Chemotoxicity induced oxidative stress occurs as a result of the reduction of foreign compounds such as insecticides, pesticides and heavy metals (Chowdhury & Saikia, 2020; Kroon et al., 2017). Environmental induced oxidative stress occurs due to ambient conditions such as dissolved oxygen, pH and water temperature, causing the production of ROS within the organism (Kroon et al., 2017). Oxidative stress and its impact can be assessed by the measurement of the reaction products of oxidative damage. Lipid peroxidation, DNA oxidation and protein oxidation are all measurable products of oxidative damage (Betteridge, 2000). The measurement of the depletion of antioxidants (used to counteract the impact of ROS) can also provide inferences of oxidative damage (Betteridge, 2000).

Heat Shock Proteins

Heat shock proteins (HSPs) are another secondary stress response that constitute a component of overall fish health. HSPs are produced when a cell is exposed to a stressor, with their production increased via changes in gene expression (Roberts et al., 2010). Contrary to their name, numerous

types of stressors such as parasites, pollution, hypoxia, ultraviolet radiation, viral infection as well as hypothermia and heat shock can induce the production of HSPs (Feder & Hofmann, 1999; Szyller & Bil-Lula, 2021). HSPs are therefore commonly used as biomarkers of exposure to these environmental stressors (Shankar & Mehendale, 2014). The physiological role of HSP's is diverse, however they are generally responsible for refolding denatured proteins, aiding in folding new proteins and deconstructing irreparable proteins. This in turn protects the cell from programmed cell death known as apoptosis and maintains homeostasis (Ponomarenko et al., 2013; Shan et al., 2020). Although this review refers separately to HSPs, they are intrinsically related to other stress responses such as oxidative stress (Szyller & Bil-Lula, 2021). Heat shock proteins can be directly measured using various laboratory techniques and in vitro studies.

Osmotic and Ion Regulation

Osmoregulation in fish is a process that primarily occurs across the gills, with the skin being relatively impermeable (Wendelaar Bonga & Lock, 1992). Osmoregulation is an important regulatory process that maintains correct concentrations of ions such as Na^+ and Cl^-, and allows for the excretion of toxicants (Evans, 1987; Wendelaar Bonga & Lock, 1992). Understanding gill anatomy is important to understanding how osmoregulatory processes can be impacted by stressors. For a comprehensive review, see that of Evans (1987). However, in brief, gill arches support filaments containing lamellae, which are covered in a thin multifunctional epithelial layer (Wendelaar Bonga & Lock, 1992). Gill lamellae are implicated as sites of ion and water diffusion (Evans, 1987; Wendelaar Bonga & Lock, 1992). Damage to the lamellar epithelium can be caused by numerous stressors such as handling and pollution, in turn increasing their permeability to water and ions (Eddy, 1981; Skomal & Mandelman, 2012; Wendelaar Bonga & Lock, 1992). This can increase energy-intensive compensatory measures to maintain water and ion homeostasis, in turn leading to reduced growth and reproduction (Wendelaar Bonga & Lock, 1992). Changes in plasma osmolality can be measured using an osmometer, and the concentration of ions can be measured using spectrophotometry (Sopinka et al., 2016). Furthermore, plasma enzymes related to ion transport and osmoregulation have been used in studies to assess responses to metal exposure (Kroon et al., 2017).

Relevant Stressors

Pottinger (2008) suggests that secondary stress responses are more likely to be activated due to prolonged or chronic activation of the HPI axis. This means that they may be more sensitive to chronic stressors such as pollution. Such is the case for HSPs, which have been recommended as bioindicators of pollution and toxins in the environment (Feder & Hofmann, 1999). Oxidative stress and the production of free-radical ROS are also influenced by the presence of environmental pollutants (Lushchak, 2011). Furthermore, changes in osmoregulatory mechanisms due to stress can occur over the course of hours and days (Eddy, 1981) and immunological suppression occurs in response to chronic stressors (Tort, 2011). The current paper did not find any papers that discussed the above secondary stress responses in the context of other environmental stressors such as hydro-ecological variability.

Strengths and weaknesses

Secondary stress responses measured in the blood can often be sampled non-lethally (Sopinka et al., 2016). Furthermore, as secondary stress responses are commonly measured in laboratory studies, equipment, assays and other resources are readily available for purchase, however this equipment or assays can be prohibitively expensive (Sopinka et al., 2016). Due to their molecular and cellular scale, requiring accurate and sophisticated equipment, the discussed secondary responses are

unlikely to be applicable as rapid field-based indicators of environmental disturbances such as hydro-ecological variability.

Tertiary Stress Response

Severe stressors will ultimately lead to changes in fish that operate on a whole-organism level. These tertiary changes are the result of maladaptations that have occurred due to allostatic overload and exhaustion of resources during the primary and secondary stress responses. Components of fish health under this classification include changes in whole-body (condition) and organ morphology (organosomatic indices), the presence of external abnormalities such as tumours and lesions, changes in behaviour and changes in lipid content (Barton et al., 2002; Barton, 2002; Madaro et al., 2020). Tertiary stress responses are considered to have broader ecological relevance when compared to primary and secondary stress responses. While primary and secondary stress responses are interrelated and interregulated with the tertiary stress response, the tertiary stress response can be considered the first interface between physiological change in a fish, its environment and its ability to perform life-history functions. For example, changes in behaviour can increase or decrease competition, while morphological changes can influence demographic processes (McCallum, 2008; McPherson, Slotte, et al., 2011).

Morphological Condition Indices

Description of methods

Condition is a broadly used term, generally encompassing approximate measures of somatic energy reserves (Gubiani et al., 2020; Kaufman et al., 2007; Labocha et al., 2014; Petitjean et al., 2020). In this section condition will refer specifically to whole-body morphological assessments based on length and weight, however the term condition in the literature can also refer to other health components such as organosomatic indices (discussed below) (Gubiani et al., 2020). In contrast to organosomatic indices, whole-body morphological assessments of fish condition are used as a surrogate measure of fat content and energy reserves (McPherson, Slotte, et al., 2011). These energy reserves represent the quantity of metabolizable tissues exceeding those required for daily nutritional demands (Brosset et al., 2015). Instead of being a direct measure, condition indices are based on the premise that fish with greater energetic status are more full-bodied, and therefore heavier at a given length (Brosset et al., 2015; Richter et al., 2000). There are numerous morphological condition indices used among the different disciplines of fisheries science, with the most popular including Fulton's K (Fulton, 1904), Le Cren's relative condition factor, K_n (Le Cren, 1951), relative weight W_r (Wege & Anderson, 1978) and length-weight regression residuals (Kaufman et al., 2007).

Fulton's K is defined as the measured weight (W) of an individual divided by its measured length (L) raised to the third exponent. Depending on the units used to measure the length and weight, Fulton's K is multiplied by a scaling factor so that the index is centred on a value of one. In the case of grams and millimetres below, the scaling factor is 100,000.

$$K = \left(\frac{W}{L^3}\right) \times 100{,}000$$

Fish with a value of 1 are considered the benchmark of condition, with scores above 1 representing fish in better condition and scores below 1 representing worse condition. Variations of Fulton's K exist, such as somatic K, which is calculated in the same manner but instead using somatic mass in order to eliminate the confounding effects of internal organs (Hards et al., 2019).

Le Cren proposed the relative condition factor in 1951. This measure, shown below, compares the weight (W) of a fish to its expected weight (W'), which is calculated using a length-weight regression derived from the population (sample specific) from which the fish was sampled (Kaufman et al., 2007).

$$Kn = (\frac{W}{W'})$$

This method also uses 1 as a benchmark of condition, where fish with a relative condition factor above 1 have a higher weight for their length compared to the average of the respective sample.

Relative weight, first proposed by Wege and Anderson (1978), is based on a similar equation to that of Le Cren's relative condition factor and is given below.

$$Wr = \left(\frac{W}{Ws}\right) \times 100$$

Here, similarly to relative condition factor, W is the weight of the fish and W_s is a length-specific standard weight calculated from a regression. The difference in this equation lies in the fact that the regression used to calculate W_s is representative of the entire geographic range of the species (species-specific), and not of a single population (Kaufman et al., 2007).

Regression residuals can also be used as an index of fish condition (Beesley et al., 2021; Kaufman et al., 2007). These are calculated from a least-square-fitted relation of length and weight (or the $\log_e$ of these). Positive residuals indicate fish in above average condition and negative residuals indicate fish in below average condition (Beesley et al., 2021).

Relevant Stressors

Values of condition that are lower than normal are an indication that stress has impacted the somatic allocation of energy (Kebus et al., 1992). As changes in condition occur slowly in a fish, it is mainly only chronic stressors that can create a marked difference in condition. Chronic stressors such as pollution, changes in hydrology, climate change, habitat degradation, crowding and competition have been shown to influence fish condition (Blackwell et al., 2000; Möllmann et al., 2005; Rennie et al., 2010; Smedley et al., 2011; Spranza & Stanley, 2000; Swingle & Shell, 1971). In particular, Swingle and Shell (1971) suggested that Relative Weight could be used to understand the impacts of pollution or any chronic stressor on lentic fish populations. Furthermore, multiple condition indices such as regression residuals (Beesley et al., 2021) and relative weight (Balcombe & Arthington, 2009) have been used in studies investigating responses to hydrological variability.

Strengths and Weaknesses

The above morphological condition indices differ in their ability to make inferences within and between fish populations. Fulton's K suffers from the assumption that all fish undergo isometric growth (growth of all parts of the fish occurs at a consistent rate), therefore maintaining a consistent shape throughout development. In many cases, fish display allometric growth, where fish proportions change due to inconsistent growth rates of different parts of the organism throughout their development (Bolger & Connolly, 1989). For example, fish becoming relatively stouter or deeper-bodied as they increase in length is an example of positive allometric growth (Riedel et al., 2007). The use of Fulton's K in the case of positive allometric growth can reduce the validity of this method, as fish become heavier than the increase in length would imply if isometric growth were occurring (Bolger & Connolly, 1989; Hards et al., 2019). In studies where assumptions of isometric

growth cannot be validated, the use of Fulton's K should be limited to fish of similar length within a population, where body proportions between fish will be similar.

Le Cren's relative condition factor removes the need for assumptions of isometric and allometric growth as it is derived from a population based length-weight regression (Lloret et al., 2013). However, as the value of W' is calculated using a length-weight relationship unique to a particular population, this method is limited in its ability to make comparisons of condition to populations which may have a different length-weight relationship to calculate W'.

Relative weight allows for the comparison of condition across populations and throughout size ranges, as W_s encompasses the length-weight relationship of all populations in that species' home range. An obvious caveat to this method is that it requires the availability of standard weight equations that represent variation across the geographical range of the species in question. Furthermore, the standard weight equations must use fish lengths that are representative of the fish length in question in order to make accurate calculations of fish condition (Lloret et al., 2013).

In addition to the relative condition factor and relative weight, regression residuals also benefit from the fact that they are not correlated with body size. This means length-weight regression residuals are not limited by assumptions of isometric or allometric growth (Kaufman et al., 2007).

Condition indices assume that changes in fat reserves result in measurable changes in morphology (Hards et al., 2019). While this has been shown to be the case in multiple species, there exists instances where fat reserves and morphological condition have been poorly correlated (for example in the work of McPherson et al. (2011) and Marshall et al. (2004)) and it would be careless to extrapolate this to all fish (Hards et al., 2019). Furthermore, changes in morphological condition may not be due to the presence of stressors, as factors like ontogeny, sexual maturation and seasonality can impact morphology (Hasler et al., 2009). Failure to account for these factors may lead to inaccurate assessments of condition (Hards et al., 2019).

To act as a meaningful surrogate of fat reserves or energy status of fish, morphometric indices should be validated against other more direct measures of fat reserves and energy status. If morphometric indices are not validated, then their ability to accurately infer fundamental condition is only putative (Bolger & Connolly, 1989; McPherson, Slotte, et al., 2011). If validated, condition indices provide an inexpensive, simple and rapid way to make inferences regarding fish health, with the potential to relate results to a range of potential anthropogenic stressors. However, the mechanisms responsible for observed declines in fish condition may vary widely, including through trophic pathways (e.g. reductions in food availability and quantity), physiological changes that affect food acquisition by fish (e.g. reductions in mobility, visual acuity) and other factors (Barton et al., 2002). Nevertheless, fish condition can be used as a way to understand how anthropogenic stressors can have broader population and community level impacts (Hards et al., 2019), as morphological condition is intrinsically related to population dynamics such as fecundity (Brosset et al., 2016), and predation pressure (Hoey & McCormick, 2004).

Organosomatic Indices

Description of Methods

While morphological condition indices are used as a surrogate measure of energetic status and fat content, organosomatic indices provide a more direct measurement of condition within a fish. Organosomatic indices compare the weight of an organ to the total body weight, with the following general equation (Hasler et al., 2009; Petitjean et al., 2020).

$$\frac{Weight\ of\ Organ}{Total\ Body\ Weight} \times 100$$

As organs are sites for lipid storage and important physiological functions, an increase in their size can indicate better nutritional status and overall better health (Dekić et al., 2016). The most common organosomatic indices are the hepatosomatic index (HSI, liver/body weight), the splenosomatic index (SSI, spleen/body weight), the gonadosomatic index (GSI, gonad/body weight) and the viscerosomatic index (VSI, entire viscera/body weight) (Barton et al., 2002).

The HSI is the most commonly used organosomatic index (Goede & Barton, 1990) and is used as a proxy for the energy content in the liver, with a larger liver generally indicating fish in better condition (Petitjean et al., 2020). Instead of being a measure of energetic status, the SSI gives an indication of hematopoietic capacity (the formation of blood cellular components) and the transfer of blood into and out of circulation. This can be used to make inferences of immunological capacity and disease resistance (Hadidi et al., 2008). An above average GSI indicates greater reproductive activity and can give insight into reproductive strategy, maturation and seasonal patterns in gonad development (Flores et al., 2014; Martínez-Gómez et al., 2012; McPherson, Ganias, et al., 2011; Rizzo & Bazzoli, 2020). Similarly to the HSI, the VSI gives insight into energetic status of fish, however Hung et al. (1997) found that the VSI responded more to starvation than the HSI.

Relevant Stressors

Organosomatic indices can give a better indication of physiological responses to stressors such as pollution, compared to more general health indices such as morphological condition (Bevelhimer et al., 2014; Van der Oost et al., 2003). Lower or higher values may indicate change in the energy allocation to organs, as a consequence of a compensatory measure (Barton et al., 2002; Hasler et al., 2009).

Strengths and Weaknesses

While organosomatic indices generally provide a reliable indication of fish health, in some cases there are confounding factors decreasing the reliability of interpretations. For example, an increase in HSI values is generally associated with increased condition and overall health, however, some studies have found that the exposure to pollutants can also cause increased HIS values through an increase in cell size and cell number (Facey et al., 2005; Martínez-Gómez et al., 2012; Porte et al., 2002; Van der Oost et al., 2003).

External abnormalities

Description of Methods

Visual assessment of external physiological anomalies is a technique often used in environmental monitoring and for rapid assessments of fish health (Tierney & Farrell, 2004). External physiological anomalies refer to any anomaly visible on a fish without the use of autopsy, and includes anomalies seen in the mouth and gill areas. Commonly recorded physiological anomalies include deformities (skeletal, eyes, appendages etc), erosion of fins, lesions, ulcers and tumours, which are often collectively referred to as DELT (Simon & Burskey, 2016). Additionally, the presence of external parasites, wounds from handling (for example from recreational or commercial fishing) and discolouration are also commonly recorded (Smith et al., 2002). Generally, these assessments can be performed with the naked eye, however correct identification of anomalies such as tumours may require microscopic pathology (Blazer et al., 2018).

Relevant Stressors

The presence of DELT anomalies has been linked to impaired water quality due to high levels of pollution (Benejam et al., 2010; Bunt & Jacobson, 2021; Simon & Burskey, 2016). Furthermore, Vethaak et al. (2009) have recorded a reduction in external skin anomalies with the improvement of ecosystem condition.

Visual assessment methods are commonly integrated into broader environmental assessments methods such as the Index of Biotic Integrity, where they are used as an indication of ecosystem condition. The presence of physiological anomalies such as parasites, lesions and tumours may be indicators that environmental stressors contributing to degraded environmental condition are also overwhelming and inhibiting the immune response of fish, necessary for maintaining their health.

Strengths and Weaknesses

External visual assessments of physiological anomalies are beneficial because they can be non-lethal, inexpensive and rapid. This makes them useful for initial assessments of a fish population, studies monitoring changes over time or in studies using a combination of fish health assessment methods (Blazer et al., 2018; Tierney & Farrell, 2004). Visual assessment with the naked eye can have limited accuracy however, as misidentification of particular anomalies can occur, for example the misidentification of a raised lesion or parasite as a tumour (Blazer et al., 2018).

Behaviour

Description of Method

Fishes display a suite of different behaviours over a range of time scales. Many of these behaviours are inherently linked to physiological processes that occur as part of the primary and secondary stress response (Barton et al., 2002). For example the release of hormones such as cortisol is associated with feeding motivation (Øverli et al., 2006), and increased allostatic load due to secondary stress responses such as changes in metabolism can lead to energetic trade-offs, reducing the aerobic scope for locomotive behaviours (Davis, 2010). Other tertiary stress responses such as reduced morphological and organosomatic condition can also impact behaviour.

Changes in behaviour can therefore indicate physiological stress and poor health on numerous biological levels (Cooke et al., 2014). The types of behaviour relevant to the assessment of fish health differs with the research question at hand. Fish behaviour can be broadly grouped as feeding, locomotion respiration, social and involuntary behaviours. For each of these main behavioural groups, there are numerous behavioural traits that can be measured for the purpose of fish health assessment (table 1).

Table 1: A summary of the types of behaviours used as stress response indicators and their respective behavioural traits.

Feeding	Locomotion	Respiration	Social	Reflexes
• Feeding motivation (Øverli et al., 2006)<br>• Feed intake<br>• Total feeding time (Martins et al., 2012).	• Swimming position<br>• Swimming speed (Jain et al., 1998).<br>• Swimming activity (Bunt & Jacobson, 2021).	• Ventilatory activity (Martins et al., 2012)<br>• Aquatic surface respiration (Bunt & Jacobson, 2021).	• Aggression (Martins et al., 2012)<br>• Schooling	• Orientation (Davis, 2010)<br>• Startle responses<br>• Fin erection<br>• Body flex upon restraint

• Foraging behaviours (Martins et al., 2012)<br>• Prey capture (Weis et al., 2001)				• Operculum and mouth clamping<br>• Gag response

Relevant Stressors

Fish behaviours, especially reflexes such as body flex and operculum and mouth clamping, are commonly measured for welfare assessment in farmed fish (Davis, 2010). These responses can act as indicators to acute stressors such as light, handling and noise (Davis, 2010). Other behavioural traits have been shown to be sensitive to environmental stressors such as contaminants. Heavy metal contaminants were implicated in a study of prey capture behaviour between fish from contaminated sites and fish in reference sites (Weis et al., 2001). The laboratory study found a strong correlation between reduced prey capture and metal accumulation in the liver (Weis et al., 2001). Prey capture is associated with fish growth, indicating that behavioural changes can be a mechanism by which fish health is reduced.

Strengths and Weaknesses

It is difficult to gain mechanistic understanding of how environmental stressors impact fish health with the use of behavioural studies alone (Cooke et al., 2014; Martins et al., 2012). Instead, the study of fish behaviour can be used as a complementary method in determining both drivers and implications of poor fish health. For example, in the discipline of conservation science, it has been suggested that studies of both behaviour (conservation behaviour) and physiology (conservation physiology) can be combined to generate new conservation solutions (Cooke et al., 2014). Similarly, physiological and behavioural responses are often measured in tandem for the assessment of stress and welfare in aquaculture (Gräns et al., 2016). Fish behaviour can be assessed alongside other health metrics such as the presence of DELT anomalies (Bunt & Jacobson, 2021). Despite their shortcomings, behavioural changes that indicate acute stressors such as those discussed above are quick and easy to observe.

Lipids and fats

Description of Method

While morphological and organosomatic condition indices are an approximate measure of the energy stores in fish tissue, the direct measurement of lipids (biochemical condition) offers a more accurate indication of energetic status (Brosset et al., 2015; Kaufman et al., 2007; Stevenson & Woods Jr, 2006). Lipids, namely triacylglycerols, are the primary energy storage material that makes up adipose tissue, commonly known as body fat (Bayly, 2014). Lipid storage and dynamics in fish are fundamental attributes of fish health. Lipid stores throughout the body supply the energetic demands of basic maintenance and metabolic needs, as well as growth and life-history functions such as reproduction (Adams, 1999). Lower stores of lipids and reduced energy status can therefore be interpreted as a reduction in health, as a fish’s ability to perform basic physiological functions is reduced. However, lipid storage is naturally variable due to both external and internal factors (Adams, 1999). Lipid accumulation is directly associated with the external environment through

trophic interactions and food availability, while physiological factors such as reproductive status can cause variability.

There are numerous direct measures of lipids in fish. Proximate body composition analysis provides an estimate of organic (bulk lipids, proteins and carbohydrates) and inorganic (water and carbon ash) compounds in the tissues of fish (Schloesser & Fabrizio, 2017). Samples for proximate body composition generally involve the homogenisation of the whole fish body and its viscera, meaning this analysis estimates total body lipid content (Kaufman et al., 2007; Schloesser & Fabrizio, 2017). Bomb calorimetry offers an estimate of the energy density of individuals by measuring the amount of heat released during combustion of a selected tissue sample (Schloesser & Fabrizio, 2017). Visual assessments of fatty deposits within the gut cavity (coelomic fat) of fishes has also been used as a condition index (Beesley et al., 2021). Assessment of individual fatty acids is another specialised form of lipid assessment, for a review of these methods see Couturier et al. (2020).

Relevant Stressors

Adams (1999) discusses that environmental stressors that necessitate the diversion of energy towards metabolic processes for maintaining and repairing damaged biological systems, would in turn reduce the allocation of energy for lipid storage. Furthermore, environmental stressors that impact food acquisition may also directly impact lipid storage. This explanation provides a mechanistic link between environmental stressors and lipid storage (Adams, 1999). Environmental stressors that could impact lipid storage include changes in pH, oxygen, siltation, contaminant levels, parasite infection and fluctuating thermal and hydrological regimes (Dehn & Schirf, 1986; Shulman & Love, 1999).

Strengths and Weaknesses

The direct measurement of lipids through proximate body composition analysis or bomb calorimetry can be lethal, time consuming and expensive, limiting their applicability in large experiments with numerous samples (Glover et al., 2010; Schloesser & Fabrizio, 2017; Wuenschel et al., 2006). Despite this, lipids have been shown to be a more sensitive indicator of stressors such as parasitic infection when compared to morphological condition estimates (Shulman & Love, 1999).

Other Health Assessment Methods

Parasites and Disease

In this section, parasitic infections have been considered separately to primary, secondary and tertiary stress responses as they can occur irrespective of physiological processes and health status. Furthermore, although assessments of parasites are often included in visual assessments of external anomalies as mentioned above, these assessments are often rudimentary presence/absence assessments that don't allow for inferences regarding environmental drivers (Crafford & Avenant-Oldewage, 2009). Therefore, in this section parasites are considered separately to provide greater insight for their use in fish health assessment in an environmental context.

Parasites are taxonomically diverse, exhibiting a variety of lifecycle strategies (Barber et al., 2000). They can occur within the body (endoparasites) as well as externally (ectoparasites) (Blanar et al., 2009). Parasites occur on multiple biological levels from single-celled organisms known as micro-parasites, to larger organisms such as isopods and nematodes known as macro-parasites (Lloret et al., 2012). The presence of parasites can either be an indication of poor fish health leading to susceptibility of infection (Marcogliese, 2004), or a driver of fish health leading to other health complications through trade-off mechanisms. As drivers of health, parasites can lead to a number of health implications such as decreased body mass, growth retardation, changes in behaviour,

necrosis, inflammation and reduced haematocrit and erythrocyte counts (Barber et al., 2000; Hansen et al., 2006). In turn this can impact the survival and population structures of host species (Marcogliese, 2004).

Relevant stressors

Parasite load in fish can act as an indicator of environmental stressors via two mechanisms. Firstly, environmental stressors can directly impact the ability of parasites to carry out functions relevant to their life history strategy, in turn reducing their occurrence in host species. Secondly, environmental stressors leading to reduced fish health can increase host susceptibility to parasitic infection (Lafferty, 1997). Any disturbances that result in increased energetic costs for fish in order to maintain their homeostasis can in turn increase susceptibility to parasites (Nanayakkara et al., 2021). Stressors that may facilitate these mechanisms have been explored in numerous reviews (Kennedy, 1997; Khan & Thulin, 1991; Lafferty, 1997; Lafferty & Kuris, 1999; Mackenzie, 1999; Overstreet, 1993; Vidal-Martinez et al., 2010), and include the presence of contaminants such as sewage and heavy metals, acidification, eutrophication and pollution (Marcogliese, 2004). When performing assessments of parasite abundance, consideration should also be given to the taxonomic groups of parasites found, as responses to natural and anthropogenic stressors have been found to be highly variable (Vidal-Martinez et al., 2010).

Strengths and Weaknesses

Although parasite loads in fish have been shown to be responsive to environmental stressors, their use for linking specific environmental stressors to reductions in fish health is limited (Vidal-Martinez et al., 2010). Potential reason for this limitation relates to the variability of parasite responses between different taxonomic groups (Lafferty, 1997). The assessment of parasite load is generally cheap and readily achievable in a field-based context, making this a suitable nonspecific indicator of environmental change (Kennedy, 1997). However the ability to relate this metric to environmental stressors will depend on the environment in which the study takes place and the number of interacting stressors (Vidal-Martinez et al., 2010).

Genetic Indices

Genetic indices of fish health primarily relate to the expression of genes as part of the stress response (Jeffries et al., 2021). Genetic approaches such as transcriptomics, which relates specifically to cellular processes at the RNA level, and genomics, relating to the DNA level, are commonly used to examine physiological status (Connon et al., 2018; Jeffries et al., 2021). In particular, ratios between RNA and DNA (Hereon referred to as RNA:DNA ratios) are one of the most widely used genetic indices. This index is based on the assumption that cellular DNA remains at a relatively constant level under changing environmental conditions, whereas the amount of cellular RNA changes due to increasing or decreasing demand for cellular expression (required for protein synthesis to enable various physiological functions) (Chícharo & Chícharo, 2008). As RNA is continuously generated from a single strand of DNA to build proteins, the ratio of RNA to DNA in the cells of fish can be used as an indicator of the amount of protein synthesis occurring, and in turn the nutritional condition of a fish (Foley et al., 2016). Fish that have been recently well-fed and are metabolically active will have a higher RNA:DNA and therefore be in better condition compared to fish that are starving and metabolically inactive (Clemmesen, 1993; Foley et al., 2016; Robinson & Ware, 1988; Suthers et al., 1996).

Relevant stressors

RNA:DNA ratios vary with food availability and abiotic environmental conditions such as water temperature and salinity (Duarte et al., 2018; Kim et al., 2008). RNA:DNA ratios have also been

employed to test nutrient-productivity models in the context of nearshore upwelling and intertidal ecosystems (Chícharo & Chícharo, 2008). Despite this, very few studies have used RNA:DNA ratios to evaluate impacts of environmental stressors on fish condition.

RNA:DNA ratios are commonly used in assessments of condition of larval fish, as they display rapid growth of purely somatic tissues, meaning RNA:DNA ratios solely indicate increased somatic growth and not other potentially confounding physiological processes such as moulting or gonadal development (Foley et al., 2016). Furthermore, larval fish can be sampled as an entire homogenate, meaning inconsistent tissue sampling is not a concern (Selleslagh & Amara, 2013). RNA:DNA ratios are still applicable for the assessment of condition in mature fish (Mathers et al., 1994), however precautions in sampling consistency must be made.

Strengths and weaknesses

Limitations to the RNA:DNA method have been discussed in terms of its high level of variability in well-fed specimens and specimens of a different age (Richard et al., 1991). Despite this, RNA:DNA ratios are frequently used as indicators of fish condition on a scale of days to weeks, meaning they can provide insights into fish nutritional status on finer time scale compared to other condition indices (Duarte et al., 2018; Foley et al., 2016).

Supplementary Figure 1

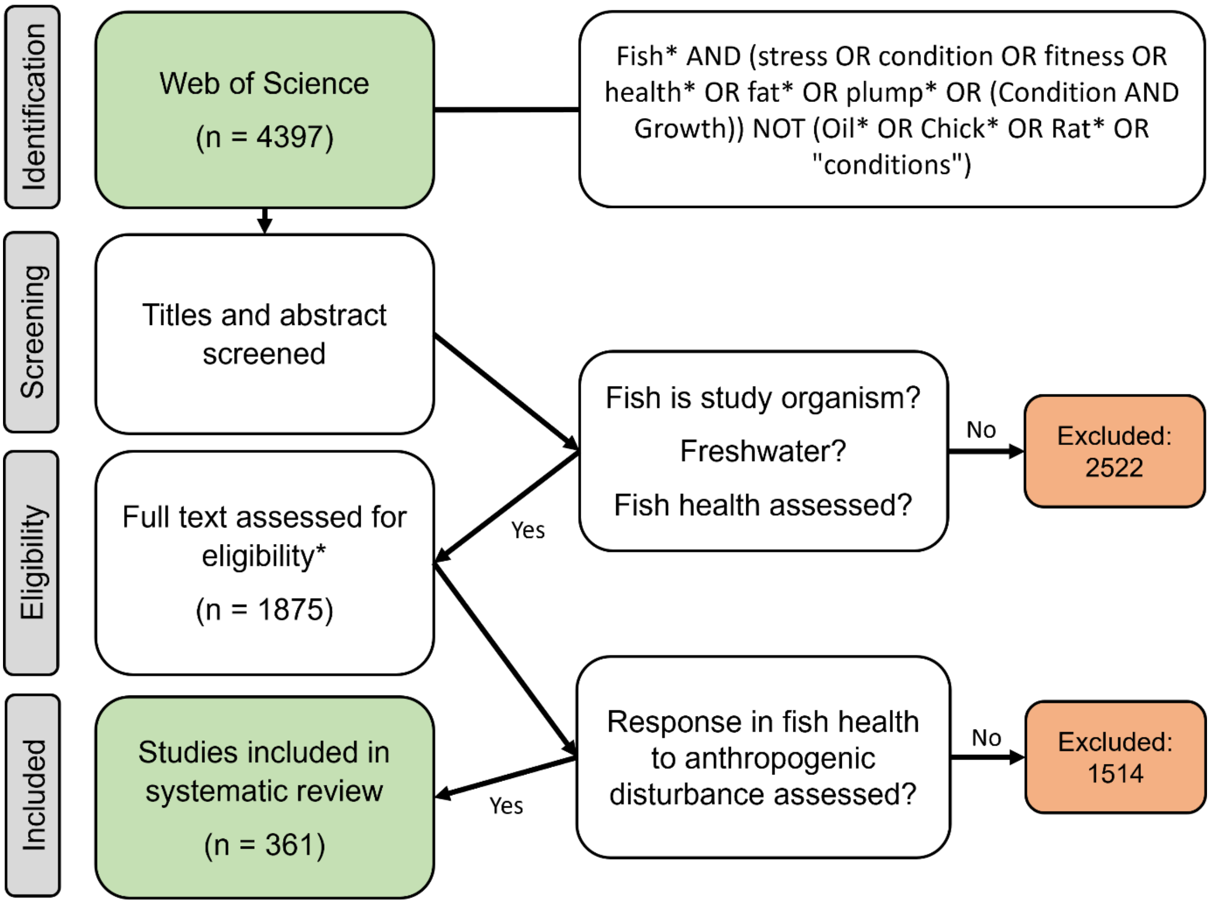

Supplementary Figure 1: Outline of review protocols in line with the Preferred Reporting Items for Systematic Reviews and Meta-Analyses (PRISMA) protocols.

Supplementary table 1

Description of stress responses used in this review

Stress response	Description
**Primary stress response**	Assessments of hormones including cortisol, dopamine, serotonin
**Secondary stress response**	Heat shock proteins, oxidative stress, haematological changes, osmolarity and ion concentrations.
**Lipids and fats (Tertiary)**	Proximate body composition analyses, whole-body lipid content, fatty acid studies.
**Behaviour (Tertiary)**	Movement and feeding activity, swimming performance, respiratory changes (e.g. operculum beat rates), startle responses, RAMP (reflex action mortality predictors)
**External abnormalities (Tertiary)**	Lesions, tumours, abrasions, deformities, fin erosion
**Organosomatic indices (Tertiary)**	Hepatosomatic index, gonadosomatic index, spleen somatic index
**Whole-body morphology (Tertiary)**	Fulton's K, relative weight, relative condition index, regression residuals,
**Parasites and disease**	Internal and external parasites, presence of diseases.
**Genetic indices**	RNA:DNA ratios
**Other**	Stomach microbiome, skin mucus, otolith chemistry

References

Adams, S. M. (1999). Ecological role of lipids in the health and success of fish populations. In *Lipids in freshwater ecosystems* (pp. 132-160). Springer.

Balcombe, S. R., & Arthington, A. H. (2009). Temporal changes in fish abundance in response to hydrological variability in a dryland floodplain river. *Marine and Freshwater Research*, *60*(2), 146-159.

Barber, I., Hoare, D., & Krause, J. (2000). Effects of parasites on fish behaviour: a review and evolutionary perspective. *Reviews in Fish Biology and Fisheries*, *10*(2), 131-165. https://doi.org/10.1023/A:1016658224470

Barton, B., Morgan, J., & Vijayan, M. (2002). Physiological and condition-related indicators of environmental stress in fish. *Biological indicators of aquatic ecosystem stress*, 111-148.

Barton, B. A. (2002). Stress in fishes: a diversity of responses with particular reference to changes in circulating corticosteroids. *Integrative and comparative biology*, *42*(3), 517-525.

Bayly, G. R. (2014). Lipids and disorders of lipoprotein metabolism. In *Clinical Biochemistry: Metabolic and Clinical Aspects* (pp. 702-736). Elsevier.

Beesley, L. S., Pusey, B. J., Douglas, M. M., Keogh, C. S., Kennard, M. J., Canham, C. A., Close, P. G., Dobbs, R. J., & Setterfield, S. A. (2021). When and where are catfish fat fish? Hydro-ecological determinants of energy reserves in the fork-tailed catfish, Neoarius graeffei, in an intermittent tropical river. *Freshwater Biology*, *66*(6), 1211-1224.

Benejam, L., Benito, J., & García-Berthou, E. (2010). Decreases in condition and fecundity of freshwater fishes in a highly polluted reservoir. *Water, Air, & Soil Pollution*, *210*(1), 231-242.

Betteridge, D. J. (2000). What is oxidative stress? *Metabolism*, *49*(2, Supplement 1), 3-8. https://doi.org/https://doi.org/10.1016/S0026-0495(00)80077-3

Bevelhimer, M. S., Adams, S. M., Fortner, A. M., Greeley, M. S., & Brandt, C. C. (2014). Using ordination and clustering techniques to assess multimetric fish health response following a coal ash spill. *Environmental toxicology and chemistry*, *33*(8), 1903-1913.

Blackwell, B. G., Brown, M. L., & Willis, D. W. (2000). Relative Weight (Wr) Status and Current Use in Fisheries Assessment and Management. *Reviews in Fisheries Science*, *8*(1), 1-44. https://doi.org/10.1080/10641260091129161

Blanar, C. A., Munkittrick, K. R., Houlahan, J., MacLatchy, D. L., & Marcogliese, D. J. (2009). Pollution and parasitism in aquatic animals: a meta-analysis of effect size. *Aquatic Toxicology*, *93*(1), 18-28.

Blazer, V. S., Walsh, H. L., Braham, R. P., & Smith, C. (2018). Necropsy-based wild fish health assessment. *Journal of visualized experiments: JoVE*(139).

Bolger, T., & Connolly, P. L. (1989). The selection of suitable indices for the measurement and analysis of fish condition. *Journal of Fish Biology*, *34*(2), 171-182. https://doi.org/10.1111/j.1095-8649.1989.tb03300.x

Breuner, C. W., Patterson, S. H., & Hahn, T. P. (2008). In search of relationships between the acute adrenocortical response and fitness. *General and Comparative Endocrinology*, *157*(3), 288-295. https://doi.org/https://doi.org/10.1016/j.ygcen.2008.05.017

Brosset, P., Fromentin, J.-M., Ménard, F., Pernet, F., Bourdeix, J.-H., Bigot, J.-L., Van Beveren, E., Roda, M. A. P., Choy, S., & Saraux, C. (2015). Measurement and analysis of small pelagic fish condition: a suitable method for rapid evaluation in the field. *Journal of Experimental Marine Biology and Ecology*, *462*, 90-97.

Brosset, P., Lloret, J., Muñoz, M., Fauvel, C., Van Beveren, E., Marques, V., Fromentin, J.-M., Ménard, F., & Saraux, C. (2016). Body reserves mediate trade-offs between life-history traits: new insights from small pelagic fish reproduction. *Royal Society Open Science*, *3*(10), 160202.

Bunt, C. M., & Jacobson, B. (2021). Exposure to a common urban pollutant affects the survival and swimming behaviour of c reek c hub (Semotilus atromaculatus). *Journal of Fish Biology*, *98*(5), 1410-1420.

Chícharo, M. A., & Chícharo, L. (2008). RNA:DNA ratio and other nucleic acid derived indices in marine ecology. *International journal of molecular sciences*, *9*(8), 1453-1471. https://doi.org/10.3390/ijms9081453
Chowdhury, S., & Saikia, S. (2020). Oxidative stress in fish: a review. *Journal of Scientific Research*, *12*(1), 145-160.
Clemmesen, C. (1993). Improvements in the fluorimetric determination of the RNA and DNA content of individual marine fish larvae. *Marine ecology progress series*, *100*, 177-183.
Connon, R. E., Jeffries, K. M., Komoroske, L. M., Todgham, A. E., & Fangue, N. A. (2018). The utility of transcriptomics in fish conservation. *Journal of Experimental Biology*, *221*(2), jeb148833.
Cooke, S. J., Blumstein, D. T., Buchholz, R., Caro, T., Fernandez-Juricic, E., Franklin, C. E., Metcalfe, J., O'Connor, C. M., St. Clair, C. C., & Sutherland, W. J. (2014). Physiology, behavior, and conservation. *Physiological and Biochemical Zoology*, *87*(1), 1-14.
Couturier, L. I., Michel, L. N., Amaro, T., Budge, S. M., Da Costa, E., De Troch, M., Di Dato, V., Fink, P., Giraldo, C., & Le Grand, F. (2020). State of art and best practices for fatty acid analysis in aquatic sciences. *ICES Journal of Marine Science*, *77*(7-8), 2375-2395.
Crafford, D., & Avenant-Oldewage, A. (2009). Application of a fish health assessment index and associated parasite index to Clarias gariepinus (Teleostei: Clariidae) in the Vaal River system, South Africa. *African Journal of Aquatic Science*, *34*(3), 261-272.
Davis, M. W. (2010). Fish stress and mortality can be predicted using reflex impairment. *Fish and Fisheries*, *11*(1), 1-11. https://doi.org/https://doi.org/10.1111/j.1467-2979.2009.00331.x
Dehn, P., & Schirf, V. (1986). Energy metabolism in largemouth bass (Micropterus floridanus salmoides) from stressed and non-stressed environments: adaptations in the secondary stress response. *Comparative Biochemistry and physiology. A, Comparative Physiology*, *84*(3), 523-528.
Dekić, R., Savić, N., Manojlović, M., Golub, D., & Pavličević, J. (2016). Condition factor and organosomatic indices of rainbow trout (Onchorhynchus mykiss, Wal.) from different brood stock. *Biotechnology in animal husbandry*, *32*(2), 229-237.
Duarte, I. A., Vasconcelos, R. P., França, S., Batista, M. I., Tanner, S., Cabral, H. N., & Fonseca, V. F. (2018). Short-term variability of fish condition and growth in estuarine and shallow coastal areas. *Marine environmental research*, *134*, 130-137. https://doi.org/https://doi.org/10.1016/j.marenvres.2018.01.008
Eddy, F. (1981). Effects of stress on osmotic and ionic regulation in fish. In (Vol. 168, pp. 77-102).
Evans, D. H. (1987). The fish gill: site of action and model for toxic effects of environmental pollutants. *Environmental health perspectives*, *71*, 47-58.
Facey, D. E., Blazer, V. S., Gasper, M. M., & Turcotte, C. L. (2005). Using fish biomarkers to monitor improvements in environmental quality. *Journal of Aquatic Animal Health*, *17*(3), 263-266.
Feder, M. E., & Hofmann, G. E. (1999). HEAT-SHOCK PROTEINS, MOLECULAR CHAPERONES, AND THE STRESS RESPONSE: Evolutionary and Ecological Physiology. *Annual Review of Physiology*, *61*(1), 243-282. https://doi.org/10.1146/annurev.physiol.61.1.243
Flodmark, L., Urke, H., Halleraker, J., Arnekleiv, J., Vøllestad, L., & Poléo, A. (2002). Cortisol and glucose responses in juvenile brown trout subjected to a fluctuating flow regime in an artificial stream. *Journal of Fish Biology*, *60*(1), 238-248.
Flores, A., Wiff, R., & Díaz, E. (2014). Using the gonadosomatic index to estimate the maturity ogive: application to Chilean hake (Merluccius gayi gayi). *ICES Journal of Marine Science*, *72*(2), 508-514. https://doi.org/10.1093/icesjms/fsu155
Foley, C. J., Bradley, D. L., & Höök, T. O. (2016). A review and assessment of the potential use of RNA: DNA ratios to assess the condition of entrained fish larvae. *Ecological Indicators*, *60*, 346-357.
Fonseca, V., Vasconcelos, R., Tanner, S., França, S., Serafim, A., Lopes, B., Company, R., Bebianno, M., Costa, M., & Cabral, H. (2015). Habitat quality of estuarine nursery grounds: Integrating non-

biological indicators and multilevel biological responses in Solea senegalensis. *Ecological Indicators*, *58*, 335-345.
Fulton, T. W. (1904). The rate of growth of fishes. *Twenty-second Annual Report*, 141-241.
Glover, D. C., DeVries, D. R., Wright, R. A., & Davis, D. A. (2010). Sample preparation techniques for determination of fish energy density via bomb calorimetry: an evaluation using largemouth bass. *Transactions of the American Fisheries Society*, *139*(3), 671-675.
Goede, R. W., & Barton, B. (1990). Organismic indices and an autopsy-based assessment as indicator of health and condition of fish. Am Fish Soc Symp,
Gräns, A., Niklasson, L., Sandblom, E., Sundell, K., Algers, B., Berg, C., Lundh, T., Axelsson, M., Sundh, H., & Kiessling, A. (2016). Stunning fish with CO2 or electricity: contradictory results on behavioural and physiological stress responses. *Animal*, *10*(2), 294-301.
Gubiani, É. A., Ruaro, R., Ribeiro, V. R., & Fé, Ú. M. G. d. S. (2020). Relative condition factor: Le Cren's legacy for fisheries science. *Acta Limnologica Brasiliensia*, *32*.
Hadidi, S., Glenney, G. W., Welch, T. J., Silverstein, J. T., & Wiens, G. D. (2008). Spleen size predicts resistance of rainbow trout to Flavobacterium psychrophilum challenge. *The Journal of Immunology*, *180*(6), 4156-4165.
Hansen, S. P., Choudhury, A., Heisey, D., Ahumada, J., Hoffnagle, T., & Cole, R. A. (2006). Experimental infection of the endangered bonytail chub (Gila elegan s) with the Asian fish tapeworm (Bothriocephalus acheilognathi): impacts on survival, growth, and condition. *Canadian Journal of Zoology*, *84*(10), 1383-1394.
Hards, A. R., Gray, M. A., Noël, S. C., & Cunjak, R. A. (2019). Utility of Condition Indices as Predictors of Lipid Content in Slimy Sculpin (Cottus cognatus). *Diversity*, *11*(5), 71. https://www.mdpi.com/1424-2818/11/5/71
Hasler, C. T., Pon, L. B., Roscoe, D. W., Mossop, B., Patterson, D. A., Hinch, S. G., & Cooke, S. J. (2009). Expanding the "toolbox" for studying the biological responses of individual fish to hydropower infrastructure and operating strategies. *Environmental Reviews*, *17*(NA), 179-197.
Hoey, A. S., & McCormick, M. I. (2004). Selective predation for low body condition at the larval-juvenile transition of a coral reef fish. *Oecologia*, *139*(1), 23-29.
Hung, S. S., Liu, W., Li, H., Storebakken, T., & Cui, Y. (1997). Effect of starvation on some morphological and biochemical parameters in white sturgeon, Acipenser transmontanus. *Aquaculture*, *151*(1-4), 357-363.
Jain, K., Birtwell, I., & Farrell, A. (1998). Repeat swimming performance of mature sockeye salmon following a brief recovery period: a proposed measure of fish health and water quality. *Canadian Journal of Zoology*, *76*(8), 1488-1496.
Jeffries, K. M., Teffer, A., Michaleski, S., Bernier, N. J., Heath, D. D., & Miller, K. M. (2021). The use of non-lethal sampling for transcriptomics to assess the physiological status of wild fishes. *Comparative Biochemistry and Physiology Part B: Biochemistry and Molecular Biology*, *256*, 110629. https://doi.org/https://doi.org/10.1016/j.cbpb.2021.110629
Kaufman, S. D., Johnston, T. A., Leggett, W. C., Moles, M. D., Casselman, J. M., & Schulte-Hostedde, A. I. (2007). Relationships Between Body Condition Indices and Proximate Composition in Adult Walleyes. *Transactions of the American Fisheries Society*, *136*(6), 1566-1576. https://doi.org/10.1577/T06-262.1
Kebus, M. J., Collins, M., Brownfield, M., Amundson, C., Kayes, T., & Malison, J. (1992). Effects of rearing density on the stress response and growth of rainbow trout. *Journal of Aquatic Animal Health*, *4*(1), 1-6.
Kennedy, C. (1997). Freshwater fish parasites and environmental quality: an overview and caution. *Parassitologia*, *39*(3), 249-254.
Kent, M. (1990). Hand-held instrument for fat/water determination in whole fish. *Food Control*, *1*(1), 47-53.

Khan, R. A., & Thulin, J. (1991). Influence of Pollution on Parasites of Aquatic Animals. In J. R. Baker & R. Muller (Eds.), *Advances in Parasitology* (Vol. 30, pp. 201-238). Academic Press. https://doi.org/https://doi.org/10.1016/S0065-308X(08)60309-7

Kim, J.-H., Kim, S.-J., Min, G.-S., & Han, K.-N. (2008). Nutritional condition determined using RNA/DNA ratios of the river pufferfish Takifugu obscurus under different salinities. *Marine ecology progress series*, *372*, 243-252.

Kroon, F., Streten, C., & Harries, S. (2017). A protocol for identifying suitable biomarkers to assess fish health: A systematic review. *PloS one*, *12*(4), e0174762.

Kumari, K., Khare, A., & Dange, S. (2014). The Applicability of Oxidative Stress Biomarkers in Assessing Chromium Induced Toxicity in the Fish <i>Labeo rohita</i>. *BioMed Research International*, *2014*, 782493. https://doi.org/10.1155/2014/782493

Labocha, M. K., Schutz, H., & Hayes, J. P. (2014). Which body condition index is best? *Oikos*, *123*(1), 111-119. https://doi.org/https://doi.org/10.1111/j.1600-0706.2013.00755.x

Lafferty, K. D. (1997). Environmental parasitology: What can parasites tell us about human impacts on the environment? *Parasitology Today*, *13*(7), 251-255. https://doi.org/https://doi.org/10.1016/S0169-4758(97)01072-7

Lafferty, K. D., & Kuris, A. M. (1999). How environmental stress affects the impacts of parasites. *Limnology and Oceanography*, *44*(3part2), 925-931.

Le Cren, E. D. (1951). The length-weight relationship and seasonal cycle in gonad weight and condition in the perch (Perca fluviatilis). *The Journal of Animal Ecology*, 201-219.

Lloret, J., Faliex, E., Shulman, G., Raga, J.-A., Sasal, P., Muñoz, M., Casadevall, M., Ahuir-Baraja, A., Montero, F., & Repullés-Albelda, A. (2012). Fish health and fisheries, implications for stock assessment and management: the Mediterranean example. *Reviews in Fisheries Science*, *20*(3), 165-180.

Lloret, J., Shulman, G., & Love, R. M. (2013). *Condition and health indicators of exploited marine fishes*. John Wiley & Sons.

Lushchak, V. I. (2011). Environmentally induced oxidative stress in aquatic animals. *Aquatic Toxicology*, *101*(1), 13-30. https://doi.org/https://doi.org/10.1016/j.aquatox.2010.10.006

Mackenzie, K. (1999). Parasites as pollution indicators in marine ecosystems: a proposed early warning system. *Marine Pollution Bulletin*, *38*(11), 955-959.

Madaro, A., Kristiansen, T. S., & Pavlidis, M. A. (2020). How Fish Cope with Stress? In T. S. Kristiansen, A. Fernö, M. A. Pavlidis, & H. van de Vis (Eds.), *The Welfare of Fish* (pp. 251-281). Springer International Publishing. https://doi.org/10.1007/978-3-030-41675-1_11

Marcogliese, D. J. (2004). Parasites: small players with crucial roles in the ecological theater. *EcoHealth*, *1*(2), 151-164.

Marshall, C. T., Needle, C. L., Yaragina, N. A., Ajiad, A. M., & Gusev, E. (2004). Deriving condition indices from standard fisheries databases and evaluating their sensitivity to variation in stored energy reserves. *Canadian Journal of Fisheries and Aquatic Sciences*, *61*(10), 1900-1917.

Martínez-Gómez, C., Fernández, B., Benedicto, J., Valdés, J., Campillo, J., León, V., & Vethaak, A. (2012). Health status of red mullets from polluted areas of the Spanish Mediterranean coast, with special reference to Portmán (SE Spain). *Marine environmental research*, *77*, 50-59.

Martins, C. I., Galhardo, L., Noble, C., Damsgård, B., Spedicato, M. T., Zupa, W., Beauchaud, M., Kulczykowska, E., Massabuau, J.-C., & Carter, T. (2012). Behavioural indicators of welfare in farmed fish. *Fish Physiology and Biochemistry*, *38*(1), 17-41.

Mathers, E., Houlihan, D., & Burren, L. (1994). RNA, DNA and protein concentrations in fed and starved herring Clupea harengus larvae. *Marine Ecology-progress Series*, *107*, 223-223.

McCallum, H. (2008). *Population parameters: estimation for ecological models* (Vol. 3). John Wiley & Sons.

McPherson, L. R., Ganias, K., & Marshall, C. T. (2011). Inaccuracies in routinely collected Atlantic herring (Clupea harengus) maturity data and correction using a gonadosomatic index model. *Journal of the Marine Biological Association of the United Kingdom*, *91*(7), 1477-1487.

McPherson, L. R., Slotte, A., Kvamme, C., Meier, S., & Marshall, C. T. (2011). Inconsistencies in measurement of fish condition: a comparison of four indices of fat reserves for Atlantic herring (Clupea harengus). *ICES Journal of Marine Science*, *68*(1), 52-60.

Möllmann, C., Kornilovs, G., Fetter, M., & Köster, F. W. (2005). Climate, zooplankton, and pelagic fish growth in the central Baltic Sea. *ICES Journal of Marine Science*, *62*(7), 1270-1280.

Nanayakkara, L., Starks, E. R., Cooper, R. N., Chow, S., Leavitt, P. R., & Wissel, B. (2021). Resource-use, body condition and parasite load metrics indicate contrasting health of stocked and native game fishes in Canadian prairie lakes. *Fisheries Management and Ecology*, *28*(1), 18-27.

Øverli, Ø., Sørensen, C., Kiessling, A., Pottinger, T. G., & Gjøen, H. M. (2006). Selection for improved stress tolerance in rainbow trout (Oncorhynchus mykiss) leads to reduced feed waste. *Aquaculture*, *261*(2), 776-781. https://doi.org/https://doi.org/10.1016/j.aquaculture.2006.08.049

Overstreet, R. M. (1993). Parasitic diseases of fishes and their relationship with toxicants and other environmental factors. *Pathobiology of marine and estuarine organisms*, *5*, 111-155.

Petitjean, Q., Jean, S., Côte, J., Larcher, T., Angelier, F., Ribout, C., Perrault, A., Laffaille, P., & Jacquin, L. (2020). Direct and indirect effects of multiple environmental stressors on fish health in human-altered rivers. *Science of The Total Environment*, *742*, 140657. https://doi.org/https://doi.org/10.1016/j.scitotenv.2020.140657

Ponomarenko, M., Stepanenko, I., & Kolchanov, N. (2013). Heat Shock Proteins. In S. Maloy & K. Hughes (Eds.), *Brenner's Encyclopedia of Genetics (Second Edition)* (pp. 402-405). Academic Press. https://doi.org/https://doi.org/10.1016/B978-0-12-374984-0.00685-9

Porte, C., Escartín, E., de la Parra, L. M. G., Biosca, X., & Albaigés, J. (2002). Assessment of coastal pollution by combined determination of chemical and biochemical markers in Mullus barbatus. *Marine ecology progress series*, *235*, 205-216.

Pottinger, T. G. (2008). The stress response in fish-mechanisms, effects and measurement. *Fish welfare*, 32-48.

Pottinger, T. G., Moran, T. A., & Morgan, J. A. W. (1994). Primary and secondary indices of stress in the progeny of rainbow trout (Oncorhynchus mykiss) selected for high and low responsiveness to stress. *Journal of Fish Biology*, *44*(1), 149-163. https://doi.org/https://doi.org/10.1111/j.1095-8649.1994.tb01591.x

Redza-Dutordoir, M., & Averill-Bates, D. A. (2016). Activation of apoptosis signalling pathways by reactive oxygen species. *Biochimica et Biophysica Acta (BBA) - Molecular Cell Research*, *1863*(12), 2977-2992. https://doi.org/https://doi.org/10.1016/j.bbamcr.2016.09.012

Rennie, M. D., Sprules, W. G., & Vaillancourt, A. (2010). Changes in fish condition and mercury vary by region, not Bythotrephes invasion: a result of climate change? *Ecography*, *33*(3), 471-482. https://doi.org/https://doi.org/10.1111/j.1600-0587.2009.06160.x

Richard, P., Bergeron, J.-P., Boulhic, M., Galois, R., & Person-Le Ruyet, J. (1991). Effect of starvation on RNA, DNA and protein content of laboratory-reared larvae and juveniles of Solea solea. *Marine ecology progress series*, 69-77.

Richter, H., Luckstadt, C., Focken, U., & Becker, K. (2000). An improved procedure to assess fish condition on the basis of length-weight relationships. *Archive of Fishery and Marine Research*, *48*(3), 255-264.

Riedel, R., Caskey, L. M., & Hurlbert, S. H. (2007). Length-weight relations and growth rates of dominant fishes of the Salton Sea: implications for predation by fish-eating birds. *Lake and Reservoir Management*, *23*(5), 528-535. https://doi.org/10.1080/07438140709354036

Rizzo, E., & Bazzoli, N. (2020). Reproduction and embryogenesis. In *Biology and Physiology of Freshwater Neotropical Fish* (pp. 287-313). Elsevier.

Roberts, R., Agius, C., Saliba, C., Bossier, P., & Sung, Y. (2010). Heat shock proteins (chaperones) in fish and shellfish and their potential role in relation to fish health: a review. *Journal of fish diseases*, *33*(10), 789-801.
Robinson, S., & Ware, D. (1988). Ontogenetic development of growth rates in larval pacific herrings, Clupea harengus pallasi, measured with RNA–DNA ratios in the strait of Georgia, British Columbia. *Canadian Journal of Fisheries and Aquatic Sciences*, *45*(8), 1422-1429.
Sadoul, B., & Geffroy, B. (2019). Measuring cortisol, the major stress hormone in fishes. *Journal of Fish Biology*, *94*(4), 540-555. https://doi.org/https://doi.org/10.1111/jfb.13904
Schloesser, R., & Fabrizio, M. C. (2016). Temporal dynamics of condition for estuarine fishes in their nursery habitats. *Marine ecology progress series*, *557*, 207-219.
Schloesser, R. W., & Fabrizio, M. C. (2017). Condition Indices as Surrogates of Energy Density and Lipid Content in Juveniles of Three Fish Species. *Transactions of the American Fisheries Society*, *146*(5), 1058-1069. https://doi.org/10.1080/00028487.2017.1324523
Schloesser, R. W., & Fabrizio, M. C. (2019). Nursery habitat quality assessed by the condition of juvenile fishes: not all estuarine areas are equal. *Estuaries and Coasts*, *42*(2), 548-566.
Schulte, P. M. (2014). What is environmental stress? Insights from fish living in a variable environment. *Journal of Experimental Biology*, *217*(1), 23-34.
Seibel, H., Baßmann, B., & Rebl, A. (2021). Blood Will Tell: What Hematological Analyses Can Reveal About Fish Welfare [Review]. *Frontiers in Veterinary Science*, *8*. https://doi.org/10.3389/fvets.2021.616955
Selleslagh, J., & Amara, R. (2013). Effect of starvation on condition and growth of juvenile plaice Pleuronectes platessa: nursery habitat quality assessment during the settlement period. *Journal of the Marine Biological Association of the United Kingdom*, *93*(2), 479-488.
Shan, Q., Ma, F., Wei, J., Li, H., Ma, H., & Sun, P. (2020). Physiological functions of heat shock proteins. *Current Protein and Peptide Science*, *21*(8), 751-760.
Shankar, K., & Mehendale, H. M. (2014). Heat-Shock Proteins. In P. Wexler (Ed.), *Encyclopedia of Toxicology (Third Edition)* (pp. 830-831). Academic Press. https://doi.org/https://doi.org/10.1016/B978-0-12-386454-3.00320-1
Sheriff, M. J., Dantzer, B., Delehanty, B., Palme, R., & Boonstra, R. (2011). Measuring stress in wildlife: techniques for quantifying glucocorticoids. *Oecologia*, *166*(4), 869-887. https://doi.org/10.1007/s00442-011-1943-y
Shulman, G., & Love, R. M. (1999). 6 - Indicators of Fish Condition. *Advances in Marine Biology*, *36*, 205-220.
Šimat, V., & Bogdanović, T. (2012). Seasonal changes in proximate composition of anchovy (Engraulis encrasicolus, L.) from the central Adriatic. *Acta Adriat*, *53*(1), 125-132.
Simon, T. P., & Burskey, J. L. (2016). Deformity, erosion, lesion, and tumor occurrence, fluctuating asymmetry, and population parameters for bluntnose minnow (Pimephales notatus) as indicators of recovering water quality in a Great Lakes Area of concern, USA. *Archives of environmental contamination and toxicology*, *70*(2), 181-191.
Skomal, G. B., & Mandelman, J. W. (2012). The physiological response to anthropogenic stressors in marine elasmobranch fishes: A review with a focus on the secondary response. *Comparative Biochemistry and Physiology Part A: Molecular & Integrative Physiology*, *162*(2), 146-155. https://doi.org/https://doi.org/10.1016/j.cbpa.2011.10.002
Smedley, R. A., Curry, R. A., & Gray, M. A. (2011). Testing the severity of Ill effects model for predicting fish abundance and condition. *North American Journal of Fisheries Management*, *31*(3), 419-426.
Smith, S. B., Donahue, A. P., Lipkin, R., Blazer, V. S., & Schmitt, C. J. (2002). *Illustrated field guide for assessing external and internal anomalies in fish*.
Sopinka, N. M., Donaldson, M. R., O'Connor, C. M., Suski, C. D., & Cooke, S. J. (2016). Stress indicators in fish. In *Fish physiology* (Vol. 35, pp. 405-462). Elsevier.

Spranza, J. J., & Stanley, E. H. (2000). Condition, growth, and reproductive styles of fishes exposed to different environmental regimes in a prairie drainage. *Environmental Biology of Fishes*, *59*(1), 99-109.

Stevenson, R., & Woods Jr, W. A. (2006). Condition indices for conservation: new uses for evolving tools. *Integrative and comparative biology*, *46*(6), 1169-1190.

Suthers, I. M., Cleary, J. J., Battaglene, S. C., & Evans, R. (1996). Relative RNA content as a measure of condition in larval and juvenile fish. *Marine and Freshwater Research*, *47*(2), 301-307.

Swingle, W. E., & Shell, E. W. (1971). Tables for computing relative conditions of some common freshwater fishes.

Szyller, J., & Bil-Lula, I. (2021). Heat shock proteins in oxidative stress and ischemia/reperfusion injury and benefits from physical exercises: A review to the current knowledge. *Oxidative Medicine and Cellular Longevity*, *2021*.

Tierney, K., & Farrell, A. (2004). The relationships between fish health, metabolic rate, swimming performance and recovery in return-run sockeye salmon, Oncorhynchus nerka (Walbaum). *Journal of fish diseases*, *27*(11), 663-671.

Tort, L. (2011). Stress and immune modulation in fish. *Developmental & Comparative Immunology*, *35*(12), 1366-1375.

Van der Oost, R., Beyer, J., & Vermeulen, N. P. (2003). Fish bioaccumulation and biomarkers in environmental risk assessment: a review. *Environmental toxicology and pharmacology*, *13*(2), 57-149.

Vethaak, A. D., Jol, J. G., & Pieters, J. P. (2009). Long-term trends in the prevalence of cancer and other major diseases among flatfish in the southeastern North Sea as indicators of changing ecosystem health. *Environmental science & technology*, *43*(6), 2151-2158.

Vidal-Martinez, V. M., Pech, D., Sures, B., Purucker, S. T., & Poulin, R. (2010). Can parasites really reveal environmental impact? *Trends in parasitology*, *26*(1), 44-51.

Wege, G. J., & Anderson, R. O. (1978). Relative weight (Wr): a new index of condition for largemouth bass. *New approaches to the management of small impoundments. American Fisheries Society, North Central Division, Special Publication*, *5*, 79-91.

Weis, J. S., Samson, J., Zhou, T., Skurnick, J., & Weis, P. (2001). Prey capture ability of mummichogs (Fundulus heteroclitus) as a behavioral biomarker for contaminants in estuarine systems. *Canadian Journal of Fisheries and Aquatic Sciences*, *58*(7), 1442-1452.

Wendelaar Bonga, S., & Lock, R. (1992). Toxicants and osmoregulation in fish.

Wendelaar Bonga, S. E. (1997). The stress response in fish. *Physiological reviews*, *77*(3), 591-625.

Weyts, F., Cohen, N., Flik, G., & Verburg-van Kemenade, B. (1999). Interactions between the immune system and the hypothalamo-pituitary-interrenal axis in fish. *Fish & Shellfish Immunology*, *9*(1), 1-20.

Woodward, J. J. (1982). Plasma catecholamines in resting rainbow trout, Salmo gairdneri Richardson, by high pressure liquid chromatography*. *Journal of Fish Biology*, *21*(4), 429-432. https://doi.org/https://doi.org/10.1111/j.1095-8649.1982.tb02848.x

Wuenschel, M. J., Jugovich, A. R., & Hare, J. A. (2006). Estimating the energy density of fish: the importance of ontogeny. *Transactions of the American Fisheries Society*, *135*(2), 379-385.
